# Influenza A virus transcription generates capped cRNAs that activate RIG-I

**DOI:** 10.1101/2024.11.12.623191

**Published:** 2024-12-03

**Authors:** Elizaveta Elshina, Emmanuelle Pitre, Marisa Mendes, Brandon Schweibenz, Rebecca L.Y. Fan, Hollie French, Ji Woo Park, Wei Wang, Leo L.M. Poon, Joseph Marcotrigiano, Alistair B. Russell, Aartjan J.W. te Velthuis

**Affiliations:** 1 Department of Molecular Biology, Princeton University, Washington Road, Princeton, NJ 08544, USA; 2 Department of Pathology, University of Cambridge, Tennis Court Road, Cambridge, CB2 1QP, United Kingdom; 3 Department of Molecular Biology, School of Biological Sciences, University of California, San Diego, 9500 Gilman Drive, La Jolla, CA 92093, USA; 4 Laboratory of Infectious Diseases, National Institute of Allergy and Infectious Diseases, NIH, Bethesda, MD 20892, USA; 5 School of Public Health, University of Hong Kong, 21 Sassoon Road, Pokfulam, Hong Kong SAR, China; 6 Lewis-Sigler Institute for Integrative Genomics, Princeton University, Washington Road, Princeton, NJ 08544, US

**Keywords:** RIG-I, influenza A virus, RNA polymerase, transcription, capped cRNA, aberrant RNA

## Abstract

During influenza A virus (IAV) infection, host pathogen receptor retinoic acid-inducible gene I (RIG-I) detects the partially complementary, 5ʹ-triphosphorylated ends of the viral genome segments and non-canonical replication products. However, it has also been suggested that innate immune responses may be triggered by viral transcription. In this study, we investigated whether an immunostimulatory RNA is produced during IAV transcription. We show that the IAV RNA polymerase can read though the polyadenylation signal during transcription termination, generating a capped complementary RNA (ccRNA), which contains the 5ʹ cap of an IAV mRNA and the 3ʹ terminus of a cRNA instead of a poly(A) tail. ccRNAs are detectable *in vitro* and in both ribonucleoprotein reconstitution assays and IAV infections. Mutations that disrupt polyadenylation enhance ccRNA synthesis and increase RIG-I-dependent innate immune activation. Notably, while ccRNA itself is not immunostimulatory, it forms a RIG-I agonist by hybridizing with a complementary negative-sense viral RNA. These findings thus identify a novel non-canonical IAV RNA species and suggest an alternative mechanism for RIG-I activation during IAV infection.

## Introduction

The innate immune response plays a pivotal role in determining the outcomes of influenza A virus (IAV) infection. Innate immune signaling can either establish a protective antiviral response or lead to a detrimental “cytokine storm” ^1,2^. While it is established that retinoic acid-inducible gene I (RIG-I) is crucial for the detection of influenza virus infection ^3–6^, the mechanisms underlying this recognition remain incompletely understood. Optimal RIG-I ligands are known to be double-stranded RNAs (dsRNA) with a 5ʹ tri- or diphosphate ^7–9^. However, during IAV infection, RIG-I is thought to bind the partially complementary, 5ʹ-triphosphorylated termini of the viral RNA (vRNA) genome segments ^10,11^. These terminal ends also serve as the promoter for IAV replication and transcription ^12^.

Non-canonical replication products, such as defective viral genomes (DVG) or mini viral RNAs (mvRNA), have also been shown to potently activate RIG-I signalling ^13–15^. Conversely, small viral RNAs (svRNA), which only contain the 5ʹ terminus of the viral promoter, do not activate RIG-I ^16^. It remains unclear, however, how the viral promoter is detected in the context of the viral ribonucleoprotein complexes (vRNP), in which the vRNA is bound by the heterotrimeric RNA polymerase, consisting of PB1, PB2 and PA subunits, and multiple copies of the viral nucleoprotein (NP) ^17,18^. Innate immune activation has also been observed when IAV replication was blocked by chemical inhibitors ^19–21^ and during infection with a single-cycle IAV incapable of replication ^22^. Under these conditions, the viral RNA polymerase is only capable of primary transcription, suggesting that IAV transcripts may contribute to innate immune activation.

Primary IAV transcription is initiated after nuclear entry of vRNPs and binding of the IAV polymerase to the serine-5 phosphorylated C-terminal domain (CTD) of a host RNA polymerase II (Pol II) ^23^. The caps of nascent Pol II mRNAs are bound by the PB2 subunit of the IAV RNA polymerase and cleaved by PA endonuclease 9–14 nt from the 5ʹ cap ^24,25^. The resulting capped oligonucleotide serves as a primer for IAV transcription initiation. Depending on the 3ʹ sequence of the capped oligonucleotide, the primer aligns either to U1 or C2 of the 3ʹ terminus of the vRNA template before the primer is elongated ^26^. Transcription termination occurs on a uridine stretch (U-stretch) located approximately 16–22 nt from the 5ʹ end of the vRNA template. When the U-stretch is in the active site, the polymerase stutters and adds a poly(A) tail to the nascent IAV mRNA instead of a sequence that is complementary to the vRNA 3ʹ terminus ^27,28^. After primary transcription and an initial round of protein synthesis, the vRNA template is replicated via a complementary RNA (cRNA) intermediate (reviewed in ^12^). New vRNA molecules can then be used for additional rounds of transcription. Importantly, the cRNA is an exact, complementary copy of the vRNA and the cRNA thus differs from the mRNA as it lacks the 5ʹ cap and contains a complete 3ʹ terminus without poly(A) tail.

Given that IAV transcription may contribute to innate immune signalling, but canonical mRNAs lack the chemistry required for RIG-I activation, we investigated whether the IAV RNA polymerase can generate a non-canonical immunostimulatory RNA during transcription. Our findings reveal that the viral polymerase can bypass the U-stretch during transcription termination, producing a capped transcript with a 3ʹ cRNA terminus, or capped cRNA (ccRNA). A T677A mutation in PB1, previously shown to enhance immune activation ^29^, exacerbates U-stretch read-through and increases ccRNA synthesis. We further show that RIG-I can detect ccRNAs when they are hybridized to a complementary vRNA or a non-canonical replication product, forming true dsRNA with a 5ʹ triphosphate. Collectively, these results identify a novel IAV RNA species that contributes to the pool of immunostimulatory non-canonical viral RNAs.

## Results

### IAV polymerase produces capped cRNAs during aberrant transcription termination

**Fig. 1|:**
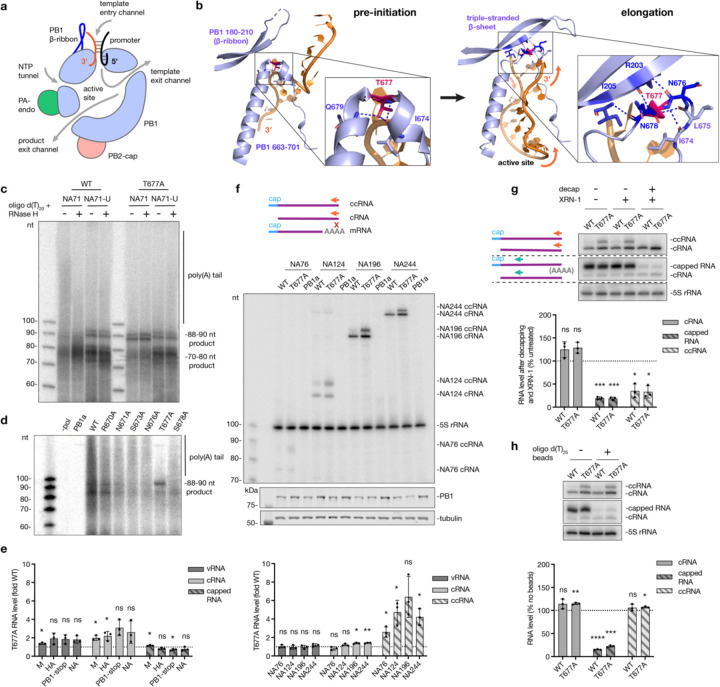
Characterization of non-canonical transcription termination and ccRNA synthesis. **a**, Schematic of the IAV RNA polymerase bound to the viral promoter. **b**, Structural comparison of the PB1 β-ribbon and PB1 residues 676–678 in the bat IAV polymerase pre-initiation (PDB: 6T0N) and elongation (PDB: 6T0V) complexes. Hydrogen bonds formed by T677 and those involved in triple-stranded β-sheet formation are indicated by blue dotted lines. T677 is colored magenta, and other residues involved in triple-stranded β-sheet formation are colored dark blue. **c**, *In vitro* transcription assay with purified wild-type or T677A polymerases on a model NA71 template or NA71 with a disrupted U-stretch (NA71-U), with and without oligo d(T)_20_-RNase H treatment. **d**, *In vitro* transcription assay with purified wild-type or mutant polymerases on a model NA71 template. Inactive polymerase mutant (PB1a) was used as a negative control. **e**, Quantification of vRNA, cRNA and all capped RNA levels produced by the T677A mutant versus wild-type polymerase in an RNP reconstitution assay with full-length templates. Primer extension gel image is provided in Supplementary Fig. 2b **f**, RNP reconstitution assay with short segment 6-based templates: schematic of primer extension for cRNA and ccRNA with a terminal cRNA primer (NA 5′) (orange, top; ‘x’ indicates no binding to the mRNA); primer extension gel and western blot analysis of PB1 expression levels (middle); quantification of vRNA, cRNA and ccRNA levels produced by the T677A mutant versus wild-type polymerase (bottom). A representative primer extension gel for vRNA image is provided in Supplementary Fig. 3b. **g**, Enzymatic digestion of total RNA from RNP reconstitution with the NA196 template: schematic of primer extension with terminal (orange) and internal (green) primers (top left); primer extension gel (top right); quantification of cRNA, all capped RNA, and ccRNA levels post-decapping and XRN-1 treatment (bottom). **h**, Oligo d(T)_25_ bead-based depletion of polyadenylated RNAs in total RNA from an RNP reconstitution assay with the NA196 template: primer extension gel with terminal (NA 5′) and internal (NA c/mRNA mini) primers (top); quantification of cRNA, all capped RNA, and ccRNA levels post-treatment (bottom). Data in panels **e**-**h** are presented as mean ± SD from three independent experiments. Statistical significance was determined using a one-sample t-test; (ns=non-significant, *P<0.05, **P<0.01, ***P<0.001, ****P<0.0001).

IAV mRNA abundance varies from <1% to >50% of all mRNAs in infected cells ^30^. The 5ʹ cap and 3ʹ poly(A) tail of IAV mRNAs are critical for preventing their detection by RIG-I and other pathogen recognition receptors ^31,32^. Since IAV transcription must start with cap-snatching, we investigated whether transcription termination can produce non-canonical transcripts. Comparison of IAV RNA polymerase structures as it transitions from pre-initiation to elongation/termination conformations showed that the promoter proximal region undergoes significant restructuring, with the PB1 β-ribbon collapsing onto PB1 residues 676–678 to form a triple-stranded β-sheet (Fig. 1a, b). PB1 T677, a highly conserved residue (Supplementary Table 1), forms dynamic interactions with the neighbouring amino acids in both conformations and thus likely plays a key role in restructuring of the PB1 β-ribbon and determining the activity of the RNA polymerase (Fig. 1b). The PB1 β-ribbon is likely a multifunctional structure, since it also contains the PB1 nuclear import signal.

To assess the role of the T677 residue in polyadenylation, we first identified polyadenylated mRNAs in an *in vitro* transcription assay using a wild-type A/WSN/33 polymerase and a segment 6-based 71 nt-long RNA (NA71), which contained an internal deletion while retaining the conserved genome termini (Supplementary Fig. 1a). We used a template of this length in order to properly resolve all products using denaturing PAGE. Following denaturing PAGE and autoradiography, we observed three distinct termination products: a prominent band that migrated between 70–80 nt of the single-stranded DNA (ssDNA) marker, a less intense double band around 88–90 nt, and a slower migrating smear (Fig. 1c, Supplementary Fig. 1b). (Note that the capped RNA products migrate slightly differently than the ssDNA marker due to the presence of additional phosphates, an additional guanine base, and methyl groups on the guanine of the cap and first base of the primer.) To verify that the slower migrating smear corresponded to transcripts containing a 3ʹ poly(A) tail, transcription reactions were treated with oligo d(T)_20_ and RNase H to specifically degrade any poly(A) tails. The oligo d(T)_20_-RNase H treatment removed the slower migrating smear and increased the intensity of the 70–80 nt band, suggesting that the latter represents mRNAs lacking the poly(A) tail (Fig. 1c, Supplementary Fig. 1b). No slower migrating smear was produced when we used an NA71 template with an interrupted U-stretch (NA71-U) that prevents polyadenylation (Fig. 1c). Interruption of the U-stretch also increased the intensity of the 88–90 nt bands (Fig. 1c). Based on the lengths of those products, we hypothesised that they originated from polymerase read-through through the 5ʹ U-stretch of the template, leading to the production of mRNAs that terminate with a cRNA 3ʹ end instead of the poly(A) tail (Supplementary Fig. 1b). The double band is produced, because the RNA polymerase can initiate on either the 3ʹ C2 or G3 of the template, as we and others described previously ^24,26^.

In contrast with the wild-type polymerase, the T677A mutation reduced polyadenylation and increased the accumulation of the 88–90 nt products (Fig. 1c, Supplementary Fig. 1c). The effect on polyadenylation was also confirmed with primer extension using a poly(A)specific primer (VNdT_20_), whereby a cDNA product of ~80 nt was observed with the wild-type polymerase but not the T677A mutant (Supplementary Fig. 1d). This product length corresponded to the combined lengths of the capped primer (11 nt), positive-sense RNA up to the polyadenylation site (50 nt), and the number of dT bases in the radiolabelled primer (20 nt) (Supplementary Fig. 1e). Polyadenylation was not affected by alanine substitutions of other PB1 residues located near the PB1 triple-stranded β-sheet (R670, K671, S673, N676 and S678) (Fig. 1d). *In vitro* activity assays on a model promoter showed that the T677A mutant was able to cleave a capped primer and initiate transcription from the 3ʹ C2 or G3 of the template with equal efficiency as the wild-type polymerase (Supplementary Fig. 1f, g), suggesting that the T677A mutation only affected transcription termination. Using RNP reconstitution assays with full-length templates we showed that the T677A mutation modestly enhanced replication (vRNA and cRNA synthesis) across most templates, but the overall levels of transcription (synthesis of all capped RNAs including mRNAs and non-canonical transcription products) either remained constant or was slightly reduced (Fig. 1e, Supplementary Fig. 2a, b).

To investigate whether the capped RNA with the 3’ cRNA terminus was produced by the IAV RNA polymerase in cell culture, we used an RNP reconstitution assay with segment 6-based templates that contained internal deletions, similar to DVGs and mvRNAs. Primer extension analysis using a primer specific for the cRNA terminal nucleotides, which are absent in mRNAs, showed two bands that migrated approximately 10–12 nt apart (Fig. 1f, Supplementary Fig. 3a). The lower band migrated at the expected size of a cRNA, and the upper band at the size of a cRNA with a 10–12 nt cap, in line with our observations from the *in vitro* assays. We will call the latter non-canonical transcription products ccRNAs from hereon. The T677A mutation increased ccRNA synthesis two- to five-fold compared to the wild-type polymerase (Fig. 1f). There was no significant effect of the T677A mutation on vRNA synthesis and an increase of cRNA synthesis was only observed for two of the four templates tested (Fig. 1f, Supplementary Fig. 3b).

To verify that the ccRNA contains a cap in cell culture, we subjected RNA to enzymatic decapping followed by XRN-1 exonuclease digestion. We measured cRNA and ccRNA levels with a cRNA-specific terminal primer and detected total capped RNA levels with an internal primer that bound to mRNA, ccRNAs and cRNA (Fig. 1g, Supplementary Fig. 4). Enzymatic treatment reduced both total capped RNA and ccRNA levels, while the cRNA levels, protected from XRN-1 digestion by the 5ʹ triphosphate group, remained unaltered (Fig. 1g). Depletion of polyadenylated RNA with oligo d(T)_25_ beads reduced capped RNA levels by 85–75%, while cRNA and ccRNA levels were not reduced (Fig. 1h). Thus, ccRNAs are capped but not polyadenylated, in line with our in vitro data.

To confirm that ccRNAs are also made on other templates, we tested their formation on short segment 5-derived templates that contained internal deletions (again, we used shorter templates to fully resolve the products using denaturing PAGE). As shown in Supplementary Fig. 3c, we observed a two-fold increase in ccRNA synthesis in reactions containing the T677A mutation. Next, we investigated if other T677 mutations affected ccRNA synthesis and found that three out of the four other amino acid substitutions tested also elevated ccRNA production (Supplementary Fig. 5a), highlighting the importance of PB1 T677 in transcription termination. Finally, we confirmed that ccRNAs were also generated by the RNA polymerases of seasonal and pandemic IAV strains in an RNP assay (Supplementary Fig. 5b). Taken together, these results confirm that IAV polymerases generate non-canonical transcripts containing a 5ʹ cap and 3ʹ cRNA terminus, and that the PB1 triple-stranded β-sheet region is likely involved in the regulation of transcription termination.

### ccRNA is produced during viral infection

**Fig. 2|:**
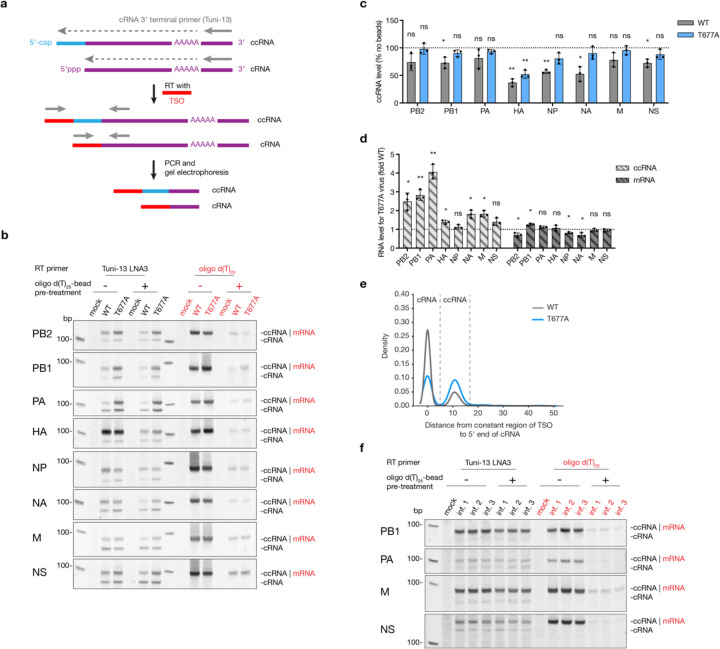
Detection of ccRNA produced during IAV infection. **a,** Schematic of TSO-based RT-PCR for analysis of 5ʹ cRNA termini. RT was done with a universal Tuni-13 or Tuni-13 LNA3 primer, which binds to the conserved 3ʹ cRNA termini of all eight segments, and in the presence of TSO. The TSO was appended by the RT enzyme to the 5ʹ termini of cDNA molecules. Subsequent PCR with a TSO-specific forward and segment-specific reverse primers was used to differentiate between cRNA with and without a 5ʹ extension. Gel electrophoresis of PCR products resolved faster-migrating bands (cRNA without 5ʹ extension) from slower-migrating bands (cRNA with a 5ʹ capped extension). **b**, Representative image of TSO-based RT-PCR analysis of infected A549 cells. Cells were infected with wild-type or T677A mutant viruses at an MOI of 0.3 or mock-infected for 24 hours. Total RNA was either treated or not with oligo d(T)_25_ beads. RT was conducted with Tuni-13 LNA3 primer (black, for cRNA and ccRNA) or an oligo d(T)_20_ primer (red, for mRNA). **c**, Quantification of ccRNA levels following oligo d(T)_25_-bead treatment. **d**, Quantification of ccRNA and mRNA synthesis by T677A versus wild-type viruses. ccRNA levels were quantified from samples post-oligo d(T)_25_-bead treatment, and mRNA levels were quantified from samples before oligo d(T)_25_-bead treatment. **e**, Analysis of 5ʹ cRNA extensions by TSO-based RT-PCR and NGS. A549 cells were infected by wild-type or T677A mutant A/WSN/33 virus at an MOI of 1 for 16 hours. The length of 5ʹ cRNA extension was expressed as a distance from the cRNA 5ʹ terminus to the TSO. The graph shows summed values for the NS segment from three biological replicates. **f**, Representative image of TSO-based RT-PCR analysis of RNA extracted 1 day post infection from three mouse lungs infected with A/Vietnam/1203/04 (H5N1) or one mock-infected with PBS. Total RNA was either depleted or not with oligo d(T)_25_ beads. RT was conducted with Tuni-13 LNA3 primer (black, for cRNA and ccRNA) or an oligo d(T)_20_ primer (red, for mRNA). Data in panels **c** and **d** are presented as mean ± SD from three independent experiments. Statistical significance was determined using a one-sample t-test; (ns=non-significant, *P<0.05, **P<0.01, ***P<0.001).

To determine if ccRNAs are generated by the IAV polymerase during infection, wild-type and T677A A/WSN/33 (H1N1) virus stocks were produced by reverse genetics and plaque purified to minimize DVG content (Supplementary Fig. 6a). The PB1 gene sequence was verified using Sanger sequencing (Supplementary Fig. 6b). Next, we employed a template switching oligonucleotide (TSO)-based RT-PCR to analyse the 5ʹ termini of the cRNA and ccRNA molecules generated in infected cells. This method uses the ability of the reverse transcriptase to append a TSO sequence to the 5ʹ termini of reverse-transcribed cRNA or ccRNA molecules, and amplify the cDNA molecules by PCR using TSO-specific forward and segment-specific reverse primers. The PCR products were subsequently separated by non-denaturing PAGE. In the gels, we observed a lower-migrating band representing the cRNA and a higher migrating product the ccRNA (Fig. 2a). To optimize the TSO-based RT-PCR and identify its limitations, we used a 196 nt-long segment 6-derived template (NA196) as a basis for the T7-based synthesis of vRNA, cRNA, ccRNA, ccRNA lacking the 25 nt of 3ʹ-terminus (ccRNA-3ʹ), and mRNA molecules. The size and quality of the products was analysed using denaturing PAGE (Supplementary Fig. 7a). The TSO-based RT-PCR effectively amplified cRNA and ccRNA molecules alone or in combination and failed to detect vRNA molecules, in line with expectations (Supplementary Fig. 7b). However, the TSO-based RT-PCR also detected ccRNA-3ʹ and mRNA molecules in the absence of cRNA molecules, indicating that mispriming during the RT step can occur in the absence of replication products. In the presence of cRNA molecules, ccRNA-3ʹ and mRNA detection was limited (Supplementary Fig. 7b). While detection of ccRNA-3ʹ molecules is likely not a concern in infection samples, a confounding mRNA signal could interfere with ccRNA detection.

To mitigate RT mispriming on mRNAs, total RNA from wild-type and T677A virus-infected samples was treated with oligo d(T)_25_ beads, which removed approximately 95% of viral mRNA as confirmed by RT-qPCR (Supplementary Fig. 8). RT-PCR analysis with a terminal cRNA-specific RT primer revealed two bands for all segments: a lower band for cRNA and upper band for ccRNA (Fig. 2b). RT-PCR analysis using an oligo d(T)_20_ RT primer showed a single band, indicative of mRNA. The oligo d(T)_20_-based depletion did not affect the cRNA levels, but significantly reduced the mRNA signal (Fig. 2b, Supplementary Fig. 8b). The ccRNA detection was also unaffected by mRNA depletion in the majority of samples, showing a similar ccRNA signal before and after treatment (Fig. 2b, c). However, for some samples and segments, the ccRNA signal was reduced after oligo d(T)_20_-based depletion, yet this reduction was much lower than the reduction for respective mRNA signal, indicating that although TSO-based RT-PCR can detect confounding mRNA molecules, the majority of the signal is derived from ccRNAs (Fig. 2b, c, Supplementary Fig. 8b). Consistent with the *in vitro* RNP assays (Fig. 1f), the T677A virus produced higher ccRNA levels than the wild-type virus for 6 of the 8 segments (Fig. 2d). The mRNA levels were comparable or reduced relative to the wild-type virus for 7 of the 8 segments (Fig. 2d). In addition, we confirmed that the T677A mutation did not increase the synthesis of non-canonical replication products, such as mvRNAs and DVGs (Supplementary Fig. 9a, b).

To validate the above findings, we performed next generation sequencing (NGS) of viral RNA from A549 cells infected with wild-type or T677A mutant A/WSN/33 (H1N1) viruses. The protocol was similar to the TSO-based RT-PCR but included an additional intra-molecular ligation step post-RT to gain NGS information on both the 5ʹ and 3ʹ end of the viral RNAs (Supplementary Fig. 10). We used this sequence information to improve our quantification of the cRNA and ccRNA levels as it allowed exclusion of any mRNA contaminants by selecting reads containing both the 5ʹ and 3ʹ cRNA termini or a 5ʹ cap and 3ʹ cRNA terminus during bioinformatic analysis. Minimal intermolecular ligation was observed. Analysis of the 5ʹ termini in circularized molecules containing a 3ʹ cRNA terminus of segment 8 revealed a bimodal distribution of the distance from the ligation site to the start of the viral genomic sequence: the first peak at 0 nt, corresponding to the 5ʹ terminus of cRNA molecules, and a second peak at 10–11 nt, corresponding to the mean length of host mRNA-derived primers forming the 5ʹ cap (Fig. 2e). The wild-type virus predominantly generated cRNA over ccRNA molecules, while the T677A virus produced similar amounts of both cRNA and ccRNA molecules (Fig. 2e). These results confirm that ccRNAs are generated by both the wild-type and T677A mutant IAV RNA polymerases during infection and that the T677A mutation increases ccRNA synthesis, in line with the data in Fig. 1.

To confirm our findings in an animal model and for a different IAV strain, we used our TSO-based RT-PCR for ccRNA detection in mice infected with the A/Vietnam/1203/04 (H5N1) virus (Supplementary Table 2). We detected ccRNAs in the lungs of infected mice for all four segments tested (Fig. 2f). No signal was detected in the PBS infected control lung. Oligo d(T)_20_-based mRNA depletion significantly reduced the mRNA signal, while ccRNA levels were only mildly reduced or not affected, indicating that the ccRNA detection was specific (Supplementary Fig. 8c). The data also reveal that ccRNA levels were particularly high for the M and NS segments compared to our analysis of A549 infections. Overall, these data show that the ccRNA synthesis occurs in infections by different IAV strains and in both in vitro and animal infection models.

### T677A mutation and ccRNA synthesis correlate with enhanced IFN responses

**Fig. 3|:**
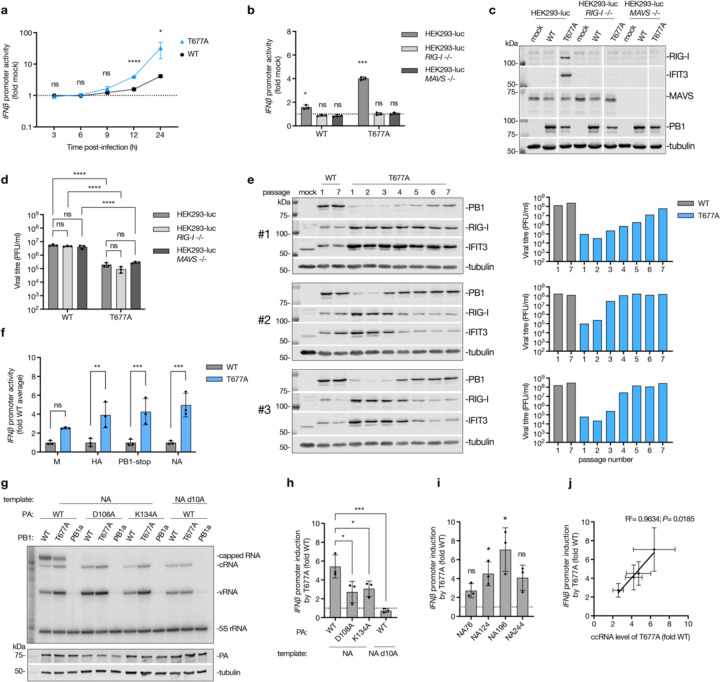
Impact of T677A mutation, transcription and ccRNA generation on IFN induction. **a**, HEK293-luc cells were infected with either wild-type or T677A mutant A/WSN/33 viruses at an MOI of 3, or mock-infected, in a synchronized infection. IFN-β luciferase reporter activity was measured at various time points post-infection. **b**, Wild-type, *RIG-I* −/− or *MAVS* −/− HEK293-luc cells were infected with wild-type or T677A mutant viruses at an MOI of 3, or mock-infected. IFN-β luciferase reporter activity was measured at 12 hours post-infection. **c**, Wild-type, *RIG-I* −/− or *MAVS* −/− HEK293-luc cells were infected with wild-type or T677A mutant viruses at an MOI of 0.01, or mock-infected for 48 hours. Protein expression was analyzed by western blotting. A representative image from three independent experiments is shown. **d**, Viral titers in cell culture supernatants collected as described in panel **c**, were quantified by plaque assay. **e**, Wild-type and T677A mutant A/WSN/33 viruses were serially passaged seven times in A549 cells at an MOI of 0.01. Supernatants and protein samples were collected 48 hours post-infection for each passage. Viral titers were determined by plaque assay, and ISG and PB1 expression levels were assessed by western blotting. Only passages 1 and 7 are shown for the wild-type virus. **f**, RNP reconstitution assay with wild-type and T677A polymerases were performed in HEK293T cells using IFN-β luciferase reporter and four different viral segments. *IFN-β* promoter activation was measured 24 hours post-transfection. **g**, RNP reconstitution assay were conducted with wild-type or T677A PB1, wild-type or transcriptionally inactive mutants of PA (D108A and K134A) and segment 6 (NA) template. The NA template lacking the 10^th^ adenosine from the 5ʹ terminus, which prevents transcription, was also tested. Primer extension was performed with internal +sense (NA 160) and –sense (NA 1280) primers, and a representative gel image from three independent experiments is shown. PA expression was analyzed by western blotting. **h**, *IFN-β* promoter activation by T677A versus wild-type polymerases in the RNP reconstitution assay described in panel g. **i**, *IFN-β* promoter activation by T677A versus wild-type polymerases in RNP reconstitution assay with short segment 6-based templates described in Fig. 1f. **j**, Correlation between *IFN-β* promoter activation and ccRNA synthesis levels from short segment 6-based templates in the RNP reconstitution assay described in Fig. 1f. Data in a, b, d, f, h-j are presented as mean ± SD from three independent experiments. Statistical significance was determined using a one-way ANOVA with Dunnett’s multiple comparisons test (a, h), two-way ANOVA with Šídák's multiple comparisons test (f) or Tukey’s multiple comparisons test (d), one-sample t-test (b, i) and simple linear regression analysis (j); (ns=non-significant, *P<0.05, **P<0.01, ***P<0.001, ****P<0.0001).

The T677A virus was previously shown to stimulate IFN responses in infected cells ^29^. To validate this observation, we used IFN-β luciferase reporter HEK293 cells (HEK293-luc) and confirmed that the T677A virus significantly elevated *IFN-β* promoter activation at 12 and 24 hours compared to the wild-type virus (Fig. 3a). To determine if the *IFN-β* promoter activation was RIG-I-dependent, we used *RIG-I* −/− or *MAVS* −/− HEK293-luc cells, in which the knock-out efficiency and IFN-β luciferase reporter functionality was verified (Supplementary Fig. 11a, b). Both the wild-type and T677A viruses failed to induce *IFN-β* promoter activity in the absence of either RIG-I or MAVS expression (Fig. 3b). Additionally, the T677A virus induced higher expression of interferon stimulated genes (ISG) (RIG-I and IFIT3) at 48 hours post-infection in wild-type HEK293-luc cells, but not in *RIG-I* −/− or *MAVS* −/− cells (Fig. 3c). Similar RIG-I-dependent ISG production was observed in A549 cells (Supplementary Fig. 12a), confirming that the T677A virus activates IFN responses via the RIG-I pathway.

The T677A virus also exhibited reduced growth in HEK293-luc and A549 cells and formed smaller plaques in MDCK cells compared to the wild-type virus (Fig. 3d; Supplementary Fig. 12b, c). The growth impairment was evident in both wild-type and immunodeficient HEK293-luc and A549 cells, indicating that the T677A mutation affects virus growth regardless of its impact on immune activation (Fig. 3d; Supplementary Fig. 12b), likely due to interference with normal polyadenylation of IAV mRNAs.

To explore potential compensatory mechanisms, we passaged T677A and wild-type viruses seven times. Those T677A virus populations where increased growth was observed were subsequently sequenced using NGS (Fig. 3e, Supplementary Table 4). For the wild-type virus, only the stock virus and passage 7 were sequenced (Supplementary Table 3). In the first T677A virus passaging replicate, the T677A mutation was conserved throughout all passages and, in spite of gradual increases in the viral titre, no change in ISG induction was observed (Fig. 3e; Supplementary Table 4). In the second and third replicate, the T677A mutation reverted to the wild-type residue by passages four and five, respectively, and this reversion coincided with a reduction in ISG production and an increase in viral titers (Fig. 3e; Supplementary Table 4). Notably, all T677A replicates showed a strong selection for an N319K mutation in NP, which was absent in the original viral stock (Supplementary Table 4). The N319 residue is surface-exposed (Supplementary Fig. 13a) and the N319K mutation was previously shown to enhance adaptation of the H7N7 avian influenza virus in mice by improving the binding of NP to importin α1 and boosting IAV replication ^33^. However, the N319K mutation did not reduce ccRNA synthesis by the wild-type or T677A mutant in an RNP assay (Supplementary Fig. 13b), which suggests that it may compensate for the effect of the T677A mutation by a different mechanism.

We next assessed if immune activation by the T677A virus is reproducible in an RNP assay. The T677A polymerase markedly increased *IFN-β* promoter activity compared to the wild-type polymerase across four full-length IAV segments (Fig. 3f). To investigate the role of transcription in T677A polymerase's ability to activate innate immune signalling, we used transcription-deficient PA mutants (K134A and D108A) ^34^ and a segment 6 template lacking the 10th adenosine (NA d10A) from the 5ʹ vRNA terminus, which abolishes transcription but not replication ^35^ (Fig. 3g; Supplementary Fig. 14). The transcription-deficient conditions significantly reduced *IFN-β* promoter induction by the T677A mutant (Fig. 3h), indicating that non-canonical transcription can contribute to innate immune activation.

In line with the above observation, the T677A polymerase also enhanced *IFN-β* promoter activity on short segment 6-based templates (Fig. 3i). Moreover, there was a positive correlation between the *IFN-β* promoter activity and the levels of ccRNAs generated on those templates (Fig. 1f, 3i–j). Consistent with this correlation, both ccRNA synthesis and *IFN-β* promoter activation were low on segment 5-based templates (Supplementary Fig. 3c). To rule out that the T677A mutation had destabilised binding to the viral promoter, making it available for RIG-I recognition, we performed promoter binding assays. T677A mutation did not affect the ability of the IAV polymerase to bind a model vRNA promoter or a full-length segment 6 (Supplementary Fig. 15a, b).

### RIG-I is activated by ccRNA in complex with negative sense viral RNA

**Fig. 4|:**
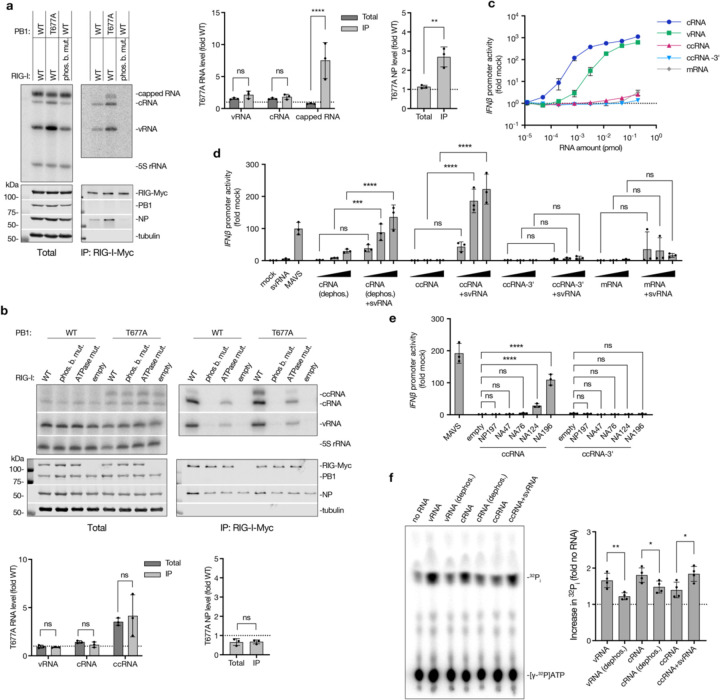
Mechanism of RIG-I activation by ccRNA. **a**, Myc-tagged RIG-I was co-expressed with wild-type or T677A mutant RNP and segment 6 (NA) template in HEK293T cells, followed by immunoprecipitation. A phosphate-binding mutant of RIG-I was used as a negative control. RNA levels in total and immunoprecipitated (IP) fractions were measured by primer extension with internal +sense (NA 160) and -sense (NA 1280) primers, and protein levels were assessed by western blotting. Shown are a representative image from three independent experiments (left) and quantification (right). **b**, Immunoprecipitation of wild-type, phosphate-binding mutant, or ATPase mutant Myc-tagged RIG-I was performed in an RNP assay with a short NA196 template. An empty plasmid served as a negative control. RNA levels were quantified by primer extension assay with terminal cRNA (NA 5′) and vRNA (NA-2) primers and protein levels assessed by western blotting. Representative image from three independent experiments (top) and quantification (bottom) are presented. **c**, HEK293-luc cells were transfected with a two-fold dilution series of *in vitro* transcribed NA196-based vRNA, cRNA, ccRNA, ccRNA-3ʹ, or mRNA, starting at 0.2 pmol. *IFN-β* promoter activity was measured 20 hours post-transfection. **d**, Increasing concentrations (0.05, 0.1 and 0.2 pmol) of *in vitro* transcribed NA196-based dephosphorylated cRNA, ccRNA, ccRNA-3ʹ and mRNA were co-transfected with 1 pmol of segment 6 svRNA into HEK293-luc cells. Total 293T RNA (mock) was used as a negative control and MAVS-encoding plasmid was used as a positive control. *IFN-β* promoter activity was measured 20 hours post-transfection. **e**, 0.2 pmol of *in vitro* transcribed NA196-based ccRNA or ccRNA-3ʹ or equivalent amount of total 293T (mock) RNA was co-transfected with pPolI plasmids encoding 47–196 nt long segment 6-based vRNA templates or segment 5-based NP197 template in to HEK293-luc cells. An empty pPolI plasmid was used as a negative control, while MAVS-encoding plasmid was used as a positive control. *IFN-β* promoter activity was measured 20 hours post-transfection. **f**, ATPase activity of recombinant RIG-I was assessed in the presence of *in vitro* transcribed NA196-based vRNA, dephosphorylated vRNA, cRNA, dephosphorylated cRNA, ccRNA or ccRNA-svRNA complex. Data in panels **a**-**f** are presented as mean ± SD from three or four independent experiments. Statistical significance was determined using two-way ANOVA with Šídák's multiple comparisons test (**a**, center; **b**, bottom left; **d**; **e**) or an unpaired two-tailed t-tests (**a**, right; **b**, bottom right; **f**) (ns=non-significant, *P<0.05, **P<0.01, ***P<0.001, ****P<0.0001).

To investigate whether ccRNA molecules produced by the IAV polymerase are recognised by RIG-I, we co-expressed Myc-tagged RIG-I or its 5ʹ phosphate-binding mutant (K861A/K858A/K851A) in an RNP assay with a full-length segment 6 template in HEK293T cells. Subsequent RIG-I immunoprecipitation and primer extension with internal primers showed increased vRNA and cRNA levels in both total and immunoprecipitated fractions in the presence of T677A mutant polymerase (Fig. 4a). Notably, there was an enrichment in viral nucleoprotein in RIG-I immunoprecipitates from T677A samples, indicating binding of an RNP containing either a full-length segment or those deletion-containing non-canonical replication products which could still form an RNP (Fig. 4a). Furthermore, there was a strong enrichment of capped RNA binding to RIG-I in the presence of the T677A polymerase, likely representing ccRNA molecules (Fig. 4a). In contrast, no significant enrichment of viral RNA or nucleoprotein levels was observed in samples containing the T677A polymerase and the segment 5 template (Supplementary Fig. 16), in line with the lower ccRNA levels produced on the short segment 5-based templates (Supplementary Fig. 3).

To test whether the segment 6-derived capped RNA detected in the RIG-I-bound fraction could have represented a ccRNA, we performed RIG-I immunoprecipitation with a short NA196 template followed by primer extension using a terminal cRNA-specific primer. ccRNA was detected in RIG-I-bound fractions in conditions with both wild-type and T677A mutant polymerases (Fig. 4b). The ccRNA levels were elevated in both the total and immunoprecipitation fractions in the presence of T677A polymerase in comparison to the wild-type polymerase. In comparison to the immunoprecipitation with the full-length NA segment (Fig. 4a), there was no enrichment in capped RNA in immunoprecipitated fraction since in this instance we specifically detected ccRNA instead of all capped RNAs (i.e., ccRNA + mRNA). The lack of nucleoprotein enrichment in the T677A condition could be due to a reduced number of NP molecules associated with the short NA196 template. Phosphate-binding and ATPase (K270A) mutations in RIG-I significantly reduced the binding of all viral RNA species, underscoring the necessity of these activities for RIG-I-mediated RNA detection, including ccRNA binding (Fig. 4b; Supplementary Fig. 17).

Given that the cap-1 structures present on IAV cap-snatched primers are incompatible with RIG-I activation ^36^, we assessed whether ccRNA molecules alone are immunostimulatory. Transfection of in vitro transcribed NA196-based RNAs (Supplementary Fig. 7a) into HEK293 IFN-β reporter cells showed that cRNA and vRNA molecules induced a strong, dose-dependent *IFN-β* promoter activation, while ccRNA, ccRNA-3ʹ, and mRNA molecules did not, suggesting that ccRNAs alone do not activate RIG-I (Fig. 4c).

We hypothesised that ccRNAs might be detected by RIG-I when their 3ʹ terminus is base-paired with a 5ʹ-triphosphorylated, complementary RNA. Such a complementary molecule could be a vRNA, DVG, mvRNA, or svRNA. To test this hypothesis, HEK293-luc cells were transfected with *in vitro* transcribed 25 nt-long segment 6 svRNA and increasing concentrations of dephosphorylated cRNA (as control), ccRNA, ccRNA-3ʹ, or mRNA (Fig. 4d, Supplementary Fig. 7a, 18). Significant *IFN-β* promoter activation occurred when svRNA molecules were combined with dephosphorylated cRNAs or ccRNAs, but not with ccRNA-3ʹ or mRNA molecules, indicating that RIG-I can detect a duplex between the 3ʹ cRNA terminus and an svRNA (Fig. 4d). Furthermore, to examine if ccRNAs could activate RIG-I with longer vRNA-like templates, we transfected ccRNA or ccRNA-3ʹ molecules into cells expressing segment 6- based vRNA templates of varying lengths or a segment 5-based template. The NA196 ccRNA induced *IFN-β* promoter activation in the presence of segment 6-based vRNA templates in a length-dependent manner, but not when the segment 5-based template was expressed (Fig. 4e). The NA196 ccRNA-3ʹ did not induce *IFN-β* promoter activation with any of the vRNA templates. The ccRNA-svRNA and ccRNA-vRNA complexes failed to induce IFN-β activation in *RIG-I* −/− HEK293-luc cells, confirming that their detection was RIG-I-dependent (Supplementary Fig. 19a, b). Finally, using purified RIG-I, we confirmed that ccRNAs can induce ATPase activity in the presence of an svRNA (Fig. 4f).

## Discussion

It is well-established that IAV genomes and non-canonical replication products are detected by RIG-I during viral infection, however the precise mechanism by which RIG-I recognises viral RNA remains poorly understood ^11,13,14^. Various lines of research indicate that RIG-I recognises the viral promoter in a ‘panhandle’ conformation formed by the partially complimentary 3ʹ and 5ʹ termini of IAV RNA species in solution in the absence of the viral polymerase ^10,11,37–39^. However, the partial complementarity of the viral promoter was shown to cause suboptimal RIG-I activation in contrast to true dsRNA ligands ^38,40^. Moreover, the IAV RNA polymerase binds the viral promoter with high affinity and the secondary structure of the polymerase-bound promoter is different from the ‘panhandle’ conformation recognised by RIG-I ^41–44^. Structural studies also suggest that the 3ʹ and 5ʹ template termini remain associated with the RNP during active transcription and replication and the nascent RNA promoter is encapsidated by a copy of polymerase as it emerges from the product exit channel ^28,45,46^. These observations suggest that RIG-I must compete with the IAV RNA polymerase or ‘wait’ for spontaneous dissociation of the RNA polymerase from the viral RNA.

The stage at which RIG-I detects viral RNA has also been a topic of debate. Although it has been proposed that RIG-I may detect incoming viral capsids ^20,47^, evidence indicates that active RNA synthesis is crucial for IAV detection ^11,15,48–51^. Experiments utilizing chemical inhibitors, which block cellular protein synthesis and viral replication, and single-cycle IAV infections, which are only capable of primary transcription, suggested that innate immune recognition may occur not only during replication, but also during transcription ^19–22^. However, delineating the contributions of viral replication versus transcription to immune activation remains a challenge. For example, Rehwinkel, et al. ^11^ showed that inhibiting transcription in the RNP assay using D108A PA endonuclease mutation did not decrease, but rather enhanced IFN production. However, this observation could also stem from an increased replication activity of the D108A mutant or the disruption of immunosuppressive interactions between PA and IRF3 ^11,52^. Furthermore, the identity of the immunostimulatory RNA generated during transcription has so far been unknown.

In this study, we identify ccRNA molecules as a species of immunostimulatory non-canonical IAV RNA produced during IAV transcription in tissue culture and mouse lungs, and we suggest a mechanism for transcription-induced RIG-I activation. Our findings demonstrate that defects in polyadenylation, specifically those induced by the T677A mutation in PB1, significantly enhance ccRNA production and RIG-I activation. Although we previously proposed that enhanced activity of the T677A polymerase might lead to the accumulation of immunostimulatory non-canonical replication products ^29^, we did not observe any increase in mvRNA and DVG synthesis by the T677A virus in this study. Instead, we observed a correlation between ccRNA synthesis induced by the T677A mutation and RIG-I activation. We propose a model in which, in addition to RIG-I activation by the viral promoter of full-length and non-canonical replication products, RIG-I may also be activated by ccRNA molecules that have hybridised with negative-sense, complementary RNAs (Supplemental Fig. 20). Future investigations are needed to determine when and where such duplexes are formed during the viral infection cycle and if they are also bound by viral proteins like NS1.

Our data also reveal that the IAV RNA polymerase produces different ccRNA steady state levels on the eight IAV vRNA segments. As with other non-canonical IAV RNAs, the potential evolutionary advantage of ccRNA production for the virus remains unclear and it is presently not clear if ccRNAs can be translated. We observed that both wild-type and the T677A mutant A/WSN/33 (H1N1) RNA polymerases generated ccRNAs in the RNP reconstitution assays and during infection. In the H5N1 infection, ccRNA:cRNA levels appeared elevated compared to the tissue culture infection, indicating that ccRNA production may vary among IAV strains and infection conditions. The ccRNA-inducing T677A mutation, however, appeared disadvantageous for the virus as it drastically reduced viral titers and the virus reverted to wild-type in two of the three passaging experiments.

The effect of the T677A mutation on polyadenylation also provides a new insight into transcription termination and into the function of the triple-stranded β-sheet, a key structural feature of the active IAV RNA polymerase and one that is less prominent in other negative sense RNA virus RNA polymerases ^28,53^. Additionally, our findings show that IAV cap-snatching and polyadenylation are not coupled enzymatic activities. It is currently unclear what factors determine whether the polymerase commits to stuttering on the U-stretch during transcription or to replicating the vRNA 5ʹ terminus during cRNA synthesis. Our results indicate that the formation of the triple-stranded β-sheet might be critical for this activity. It has also been previously observed that mutations in the B-site for 3ʹ vRNA template binding can result in premature transcription termination ^54^, but remains to be determined whether the B-site is also involved in polyadenylation. The efficiency of polyadenylation appears to be template-dependent as we observe differences in the levels of ccRNA synthesis induced by the T677A mutation from segment 5- and segment 6-based model templates and between viral genomic segments during an infection. Our experiments provide the tools to study the implication of these differences for viral infection.

Recent discoveries of svRNAs, mvRNAs, and now ccRNAs, suggest that the repertoire of non-canonical IAV RNAs may be far more extensive and complex in their interactions than previously assumed. Further exploration of the non-canonical RNA diversity, along with their roles in viral life cycle and pathogenesis, may be essential for our understanding of influenza disease and the development of more effective antiviral strategies.

## Supplementary Material

Supplement 1

Supplement 2

Supplement 3

## Data Availability

The raw gel data that support the findings of this study are available from the corresponding author upon request. NGS data from virus passaging have been deposited in the NCBI database under the accession code PRJNA1177455. NGS data from TSO-based RT PCR have been deposited in the NCBI database under the accession code GSE276698.
