# Supplementary material for: Influenza A virus transcription generates capped cRNAs that activate RIG-I
Source: bioRxiv. 2024 Dec 3:2024.11.12.623191. Originally published 2024 Nov 12. Preprint. [Version 2] doi: 10.1101/2024.11.12.623191 (PMC11601390; doi:10.1101/2024.11.12.623191)
Supplement: Supplement 1 [file media-1.pdf]

Supplementary Tables

Influenza A virus transcription generates capped cRNAs that activate RIG-I

Elizaveta Elshina[1,2], Emmanuelle Pitre[1,2], Marisa Mendes[3], Brandon Schweibenz[4], Rebecca L.Y. Fan[5], Hollie French[2,#], Ji Woo Park[1], Wei Wang[6], Leo L.M. Poon[5], Joseph Marcotrigiano[4], Alistair B. Russell[3], Aartjan J.W. te Velthuis[1,*]

Supplementary Table 1. Amino acid variation at position 677 of IAV PB1 (analysis done using www.fludb.org database).

RESIDUE 677	NO. OF STRAINS (% CONSERVATION)
T	45,293 (99.98)
S	3 (0.01)
M	1 (0.00)
A	1 (0.00)
P	3 (0.01)

Supplementary Table 2.

MOUSE	INOCULATION	WEIGHT DAY 0	WEIGHT DAY 1	VIRUS TITRE (PFU/ML)
Mock	PBS	20.20 g (100%)	19.75 g (97.77%)	<100
Infected 1	A/Vietnam/1203/04 (H5N1)	19.90 g (100%)	18.75 g (94.22%)	1.60E+05
Infected 2	A/Vietnam/1203/04 (H5N1)	18.60 g (100%)	17.83 g (95.86%)	2.70E+05
Infected 3	A/Vietnam/1203/04 (H5N1)	20.30 g (100%)	19.14 g (94.29%)	2.10E+05

Supplementary Table 3. Variants identified in the stock and passages of the wild-type A/WSN/33 virus.

SEGMENT	POSITION	TYPE	REFERENCE	ALLELE	AMINO ACID/ORF CHANGE	VARIANT FREQUENCY (%)			
						**stock**	**P7 (1)**	**P7 (2)**	**P7 (3)**
**3 (PA)**	1711	snp	T	C	silent	99	99	99	99
**6 (NA)**	66	snp	T	C	V16A	82			
**5 (NP)**	143	snp	T	C	I33T	74	36	35	27
**6 (NA)**	1368	snp	C	G	T450S		100	100	100
**6 (NA)**	1103	snp	A	G	N362D		82	77	82
**6 (NA)**	1325	snp	A	G	T436A		78	78	71
**6 (NA)**	1330	snp	A	G	silent		77	78	71
**3 (PA)**	244	del	CACAGATTTGAAATA	CA	frameshift (81 aa product)		30		
**3 (PA)**	166	del	TATTCA	TA	stop (47 aa product)		26		
**3 (PA)**	51	del	CAATC	CATC	frameshift (11 aa product)		26		
**3 (PA)**	1943	del	TATTGGCAAAGTCGGTATT	TA	del 641-651		15		

			CAACAGCTTGTATGCA				
**1 (PB2)**	2300	snp	T	C	I760T	14	
**2 (PB1)**	1882	del	TACCAGGGGCGTTTATGCA	TA	stop (619 aa product)		14

Supplementary Table 4. Variants identified in the stock and passages of the T677A A/WSN/33 virus.

SEGMENT	POSITION	TYPE	REFERENCE	ALLELE	AMINO ACID/ ORF CHANGE	VARIANT FREQUENCY (%)						
						**stock**	**P5 (1)**	**P7 (1)**	**P3 (2)**	**P4 (2)**	**P4 (3)**	**P5 (3)**
**2 (PB1)**	2053	snp	A	G	T677A	100	99	100	13	9	14	8
**5 (NP)**	1002	snp	T	G	N319K		67	80	24	47	22	45
**2 (PB1)**	1650	snp	A	T	silent		34	73				
**2 (PB1)**	776	snp	T	G	F251C		33	71				
**5 (NP)**	1175	snp	G	A	S377N		19	14				
**4 (HA)**	1583	snp	T	A	D517E		18	21				
**1 (PB2)**	123	snp	G	A	silent		18	21				
**5 (NP)**	37	snp	A	G	non-coding region		16	12				
**2 (PB1)**	516	snp	A	G	I164M		14	11				
**4 (HA)**	1136	snp	T	C	silent		10	7				
**4 (HA)**	1160	snp	C	A	silent				58	60		
**8 (NS)**	553	snp	A	T	N176I (NS1) M19L (NEP)				23	22	40	35
**5 (NP)**	1450	snp	G	A	E469K				15	16	13	12
**2 (PB1)**	2226	snp	G	A	silent				15	10	11	12
**4 (HA)**	116	snp	C	T	silent				15	12	14	13
**1 (PB2)**	2226	snp	G	A	silent				11	9	9	12
**4 (HA)**	1660	snp	T	A	stop (542 aa product)				10	11	10	11
**5 (NP)**	711	snp	G	A	M222I						9	11
**3 (PA)**	243	del	GCAC	GC	frameshift (75 aa product)						9	24
**3 (PA)**	115	del	GAAACAAAC AAAT	GAAACA AAT	frameshift (59 aa product)						7	22

Supplementary Table 5. Primers used for site-directed mutagenesis.

PRIMER NAME	MUTATION	PRIMER SEQUENCE (5′- 3′)
PB1 R670A Fw	PB1 R670A	CTCCTGGATCCCCAAAGCAAATCGATCCATCTTG
PB1 R670A Rev	PB1 R670A	CAAGATGGATCGATTTGCTTTGGGGATCCAGGAG
PB1 N671A Fw	PB1 N671A	GGATCCCCAAAAGAGCTCGATCCATCTTGAATACAAGC
PB1 N671A Rev	PB1 N671A	GCTTGTATTCAAGATGGATCGAGCTCTTTTGGGGATCC
PB1 S673A Fw	PB1 S673A	GGATCCCCAAAAGAAATCGAGCTATCTTGAATACAAGCC
PB1 S673A Rev	PB1 S673A	GGCTTGTATTCAAGATAGCTCGATTTCTTTTGGGGATCC
PB1 N676A Fw	PB1 N676A	GGATCCCCAAAAGAGCTCGATCCATCTTGAATAC
PB1 N676A Rev	PB1 N676A	GTATTCAAGATGGATCGAGCTCTTTTGGGGATCC
PB1 T677A Fw	PB1 T677A	CATCTTGAATGCAAGCCAAAG
PB1 T677A Rev	PB1 T677A	GATCGATTTCTTTTGGGG
PB1 S678A Fw	PB1 S678A	CGATCCATCTTGAATACAGCTCAAAGAGGAATACTTG
PB1 S678A Rev	PB1 S678A	CAAGTATTCCTCTTTGAGCTGTATTCAAGATGGATCG
NP N319K Fw	NP N319K	CAGCCTAATCAGACCAAAGGAGAATCCAGCACACAAG
NP N319K Rev	NP N319K	CTTGTGTGCTGGATTCTCCTTTGGTCTGATTAGGCTG
RIG-I K270A Fw	RIG-I K270A	CTACAGGTTGTGGAGCAACCTTTGTTTCAC
RIG-I K270A Rev	RIG-I K270A	GTGAAACAAAGGTTGCTCCACAACCTGTAG
RIG-I K861A/K858A/K851A	RIG-I K861A/K858A/K851A	AAGTTTTGAAGCAAGAGCAGCGATATTCTGTGCCCGACAGAACTGC
RIG-I K861A/K858A/K851A	RIG-I K861A/K858A/K851A	GAAAACTGCGCTGGCTTGGGATGTGGTCTACTCACAAAGCATTCC

Supplementary Table 6. Sequences of internally truncated vRNA templates used in RNP assays.

TEMPLATE NAME	VRNA-SENSE SEQUENCE (5′- 3′)
NA76	AGUAGAAACAAGGAGUUUUUUGAACAAACUACUUGUCAAUUUCUGGUUUGGAUUCAUUUAAACUCCUGCUUUUGCU

NA124	AGUAGAAACAAGGAGUUUUUUGAACAAACUACUUGUCAAUGGUGAACGGGAGCUCAGCACCGUACAGAUCGAUCCAAUGGUUAUUAUUUUCUGGUUUGGAUUCAUUUAAACUCCUGCUUUUGCU
NA196	AGUAGAAACAAGGAGUUUUUUGAACAAACUACUUGUCAAUGGUGAACGGGAGCUCAGCACCGUCUGGCCAAGACCAAUCUACAGUAUCACCAUUCACACUAUUUGCAAUAUUAGGCUAAUUAUUCCGACUACCAUACAGAUCGAUCCAAUGGUUAUUAUUUUCUGGUUUGGAUUCAUUUAAACUCCUGCUUUUGCU
NA244	AGUAGAAACAAGGAGUUUUUUGAACAAACUACUUGUCAAUGGUGAACGGGAGCUCAGCACCGUCUGGCCAAGACCAAUCUACAGUAUCACCAUUCACACCACAAAAAGAAAUGAUGCUCCCACAAUCCAUAUUGAGAUUAUAUUUCCUAUUUGCAAUAUUAGGCUAAUUAUUCCGACUACCAUACAGAUCGAUCCAAUGGUUAUUAUUUUCUGGUUUGGAUUCAUUUAAACUCCUGCUUUUGCU
NP76	AGUAGAAACAAGGGUAUUUUUCUUUACUAGUGACUUCGAUGUCACUCUGUGAGUACUAGUCUACCCUGCUUUUGCU
NP125	AGUAGAAACAAGGGUAUUUUUCUUUAAUUGUCGUACUCCUCUGCAUUGUCACUAGUUCGUUUGGUGCCUUUGGUCGCCAUGAUUUCGAUGUCACUCUGUGAGUACUAGUCUACCCUGCUUUUGCU
NP197	AGUAGAAACAAGGGUAUUUUUCUUUAAUUGUCGUACUCCUCUGCAUUGUCUCCGAAGAAAUAAGAUCCUUCAUUACUCAUGUCAAAGGAGGGCACGAUCGGGCUCGUUGCACUAGUCUGUUCGUAAGAUCGUUUGGUGCCUUUGGUCGCCAUGAUUUCGAUGUCACUCUGUGAGUACUAGUCUACCCUGCUUUUGCU
NP246	AGUAGAAACAAGGGUAUUUUUCUUUAAUUGUCGUACUCCUCUGCAUUGUCUCCGAAGAAAUAAGAUCCUUCAUUACUCAUGUCAAAGGAGGGCACGAUCGGGCUCGUUGCCUUUUCGUCCGAGAGCUCGAAGACUCCCCGCCCCUGGAAAGACACUAGUCUCCAUCUGUUCGUAAGAUCGUUUGGUGCCUUUGGUCGCCAUGAUUUCGAUGUCACUCUGUGAGUACUAGUCUACCCUGCUUUUGCU

Supplementary Table 7. Primers used for primer extension.

PRIMER NAME	TARGET RNA	PRIMER SEQUENCE (5′- 3′)
5S 100	5S rRNA	TCCCAGGCGGTCTCCCATCC
NP 149-	NP vRNA	ATTTCTTCGGAGACAATGCAG
NP 149+	NP c/mRNA internal	TAAGATCGTTTGGTGCCTTTG
NA 1280	NA vRNA internal	TGGACTAGTGGGAGCAT
NA 160	NA c/mRNA internal	TCCAGTATGGTTTTGATTTCC
PB1 vRNA	PB1 vRNA internal	TGATTTCGAATCTGGAAGGA
PB1 c/mRNA	PB1 c/mRNA internal	TCCATGGTGTATCCTGTTCC
HA vRNA	HA vRNA internal	TACTCAACTGTCGCCAGTTC

HA c/mRNA	HA c/mRNA internal	GTCACTGCCACATTCTTCTC
M vRNA	M vRNA internal	GAAAAGAGGGCCTTCTACGG
M c/mRNA	M c/mRNA internal	AGCCATTCCATGAGAACCTC
NA 5′	NA cRNA terminal	AGTAGAAACAAGGAGTTTTTTG
NA-2	NA vRNA terminal	AGCGAAAGCAGGAGTTTAAATG
NA c/mRNA mini	NA c/mRNA internal (for short templates)	CTACTTGTCAATGGTGAACG
NP 5′	NP cRNA terminal	AGTAGAAACAAGGGTATTTTTC
GC-	NP vRNA terminal	AGCAAAAGCAGGGTAGACTAGT
NP c/mRNA mini	NP c/mRNA internal (for short templates)	GTCGTACTCCTCTGCATTG
$VNdT_{20}$	Polyadenylated RNA	VNTTTTTTTTTTTTTTTTTTTT

V for Adenine, Guanine or Cytosine
N for Adenine, Guanine, Cytosine or Thymine

Supplementary Table 8. RNA templates used for polymerase activity assays.

NAME	SEQUENCE (5′- 3′)
AG11_primer for capping	ppGAAUACUCAAG
vRNA_5p	AGUAGAAACAAGGCC
vRNA_3p	GGCCUGCUUUUGCU
NA71	AGUAGAAACAAGGAGUUUUUUGAACAAACUACUUGUCAAUGGUUGGAUUCAUUUAAACUCCUGCUUUUGCU
NA71-U	AGUAGAAACAAGGAGAUGUGUGAACAAACUACUUGUCAAUGGUUGGAUUCAUUUAAACUCCUGCUUUUGCU
NP71	AGUAGAAACAAGGGUAUUUUUCUUUACUAGUUAGGUAGUAUACCUAGUAACUAGUCUACCCUGCUUUUGCU
NP71-U	AGUAGAAACAAGGGAAUGUGUCUUUACUAGUUAGGUAGUAUACCUAGUAACUAGUCUACCCUGCUUUUGCU

Supplementary Table 9. Primers used for mvRNA and 5S rRNA RT-PCR.

PRIMER	TARGET	STEP	PRIMER SEQUENCE (5′- 3′)

Lv3aa	mvRNA	RT	GTTCAGACGTGTGCTCTTCCGATCTAGC+A+AAAGCAGG
Lv3ga	mvRNA	RT	GTTCAGACGTGTGCTCTTCCGATCTAGCG+AAAGCAGG
Lv5	mvRNA	2nd strand	CACGACGCTCTTCCGATCTHNNNNNNNAGTAGAA+A+CAAGG
P7	mvRNA	PCR	GACGTGTGCTCTTCCGATCT
P5_IRdye800	mvRNA	PCR	CACGACGCTCTTCCGATCT
5S 100	5S rRNA	RT	TCCCAGGCGGTCTCCCATCC
5S_Fw	5S rRNA	2nd strand and PCR	GTCTACGGCCATACCACC
5S100_Rev_ATTO647N	5S rRNA	PCR	TCCCAGGCGGTCTCCCATCC

+ for LNA bases

H for Adenine, Cytosine or Thymine

N for Adenine, Guanine, Cytosine or Thymine

Supplementary Table 10. Primers/oligos used for TSO-based RT-PCR and qPCR.

PRIMER/OLIGO	TARGET	VIRUS STRAIN	STEP	PRIMER SEQUENCE (5′- 3′)
Tuni-13	cRNA, ccRNA	All	RT	ACGCGTGATCAGTAGAAACAAGG
Tuni-13 LNA3	cRNA, ccRNA	All	RT	ACGCGTGATCAGTAGAAA+CA+AG+G
Oligo $d(T)_{20}$	mRNA	All	RT	TTTTTTTTTTTTTTTTTTTT
TSO	N/A	N/A	RT	GCTAATCATTGCAAGCAGTGGTATCAACGCAGAGTACATrGrGrG
TSO Fw	TSO	N/A	PCR	CATTGCAAGCAGTGGTATCAAC
PB2 Rev	PB2	A/WSN/33	PCR	CTGCGACATTAGATTCCTTAGTTC
PB1 Rev	PB1	A/WSN/33, A/Vietnam/1203/04	PCR	GTAAAGTCGGATTGACATCCATTC
PA Rev	PA	A/WSN/33, A/Vietnam/1203/04	PCR	GGATTGAAGCATTGTCGCAC
HA Rev	HA	A/WSN/33	PCR	CAGGACTAGTACAAAAGCCTTC
NP Rev	NP	A/WSN/33	PCR	GATTTCGATGTCACTCTGTGAG
NA Rev	NA	A/WSN/33	PCR	GATCCAATGGTTATTATTTTCTGGTTTG
M Rev	M	A/WSN/33, A/Vietnam/1203/04	PCR	GACCTCGGTTAGAAGACTCATC

NS Rev	NS	A/WSN/33	PCR	GCTTGACACAGTGTTTGGATC
NS H5N1 Rev	NS	A/Vietnam/1203/04	PCR	GCGGACATGCCAAAGAAAG
18S rRNA Fw	18S rRNA	N/A	qPCR	ACCCGTTGAACCCCATTGGTGA
18s rRNA Rev	18S rRNA	N/A	RT/qPCR	GCCTCACTAAACCATCCAATCGG
NA qPCR Fw	NA	A/WSN/33	qPCR	GTTTGAATCGGTTGCTTGG
NA qPCR Rev	NA	A/WSN/33	qPCR	CTGCTCCATCATCTGGACC

+ for LNA bases
r for ribonucleotides

Supplementary Table 11. Primers/oligos used for TSO-based RT-PCR and NGS library preparation.

PRIMER/OLIGO	TARGET	STEP	PRIMER SEQUENCE (5′- 3′)
3′ cRNA primer	cRNA, ccRNA	RT	ATATGGTCTCGTATTAGTAGAAACAAGG
TSO 2	N/A	RT	Biotin-AAGCAGTGGTATCAACGCAGAGTACATrNrG+G
TSO-specific circularization primer	TSO	PCR of 5′ region	AATACGAGACCATATAAGCAGTGGTATCAACGCAGAGT
Ba-PB2-2341R	PB2	PCR of 5′ region	ATATGGTCTCGTATTAGTAGAAACAAGGTCGTTT
Bm-PB1-2341R	PB1	PCR of 5′ region	ATATGGTCTCGTATTAGTAGAAACAAGGCATTT
Bm-PA-2233R	PA	PCR of 5′ region	ATATGGTCTCGTATTAGTAGAAACAAGGTACTT
Bm-NS-890R	HA/NS	PCR of 5′ region	ATATGGTCTCGTATTAGTAGAAACAAGGGTGTTTT
Bm-NP-1565R	NP	PCR of 5′ region	ATATGGTCTCGTATTAGTAGAAACAAGGGTATTTTT
Ba-NA-1413R	NA	PCR of 5′ region	ATATGGTCTCGTATT AGTAGAAACAAGGAGTTTTTT
Bm-M-1027R	M	PCR of 5′ region	ATATGGTCTCGTATTAGTAGAAACAAGGTAGTTTTT
NSrnd1UP	NS	NS PCR	TGCCTCATCAGATTCTTCCTTC
NSrnd1DWN	NS	NS PCR	GCAGTAATGAGAATGGGAGACC
NSrnd2UP	NS	Append partial Illumina adapter	TCGTCGGCAGCGTCAGATGTGTATAAGAGACAGAGTGCTGCCTCTTCCTCTTA
NSrnd2DWN	NS	Append partial Illumina adapter	GTCTCGTGGGCTCGGAGATGTGTATAAGAGACAGGGAACAATTAGGTCAGAAGTTTGAGG

+ for LNA bases

r for ribonucleotides

N for Adenine, Guanine, Cytosine or Thymine

Supplementary Table 12. Primers/oligos used for *in vitro* transcription.

PRIMER/OLIGO	PRODUCT	PRIMER SEQUENCE (5′- 3′)
NA T7 ccRNA/mRNA Fw	ccRNA/mRNA	TAATACGACTCACTATAGGGAATACTCAAGGCAAAAGCAGGAGTTTAAATG
NA T7 vRNA/svRNA Fw	vRNA/svRNA	TAATACGACTCACTATTAGTAGAAACAAGGAGTTTTTTGAAC
NA T7 cRNA Fw	cRNA	TAATACGACTCACTATTAGCAAAAGCAGGAGTTTAAATG
NA cRNA/ccRNA rev (NA 5′)	cRNA/ccRNA	AGTAGAAACAAGGAGTTTTTTG
NA mRNA Rev	mRNA	TTTTTTTTTTTTTTTTTTTTTTTTTTTTTTGAACAAACTACTTGTCAATGGTG
NA ccRNA-3′ Rev	ccRNA-3′	AAACTACTTGTCAATGGTGAACG
NA vRNA Rev	vRNA	AGCAAAAGCAGGAGTTTAAATG
NA svRNA rev	svRNA	GTTCAAAAAACTCCTTGTTTCTACTAATAGTGAGTCGTATTA

Supplementary Table 13. Sequences of *in vitro* transcription products.

PRODUCT NAME	SEQUENCE (5′- 3′)
NA196 vRNA	AGUAGAAACAAGGAGUUUUUUGAACAAACUACUUGUCAAUGGUGAACGGGAGCUCAGCACCGUCUGGCCAAGACCAAUC UACAGUAUCACCAUUCACACUAUUUGCAAUAUUAGGCUAAUUAUUCCGACUACCAUACAGAUCGAUCCAAUGGUUAUUA UUUUCUGGUUUGGAUUCAUUUAAACUCCUGCUUUUGCU
NA196 cRNA	AGCAAAAGCAGGAGUUUAAAUGAAUCCAAACCAGAAAAUAAUAACCAUUGGAUCGAUCUGUAUGGUAGUCGGAAUAAU UAGCCUAAUAUUGCAAAUAGUGUGAAUGGUGAUACUGUAGAUUGGUCUUGGCCAGACGGUGCUGAGCUCCCGUUCAC CAUUGACAAGUAGUUUGUUCAAAAAACUCCUUGUUUCUACU
NA196 mRNA	GGGAAUACUCAAGGCAAAAGCAGGAGUUUAAAUGAAUCCAAACCAGAAAAUAAUAACCAUUGGAUCGAUCUGUAUGGUA GUCGGAAUAAUUAGCCUAAUAUUGCAAAUAGUGUGAAUGGUGAUACUGUAGAUUGGUCUUGGCCAGACGGUGCUGAGC UCCCGUUCACCAUUGACAAGUAGUUUGUUCAAAAAAAAAAAAAAAAAAAAAAAAAAAAAA
NA196 ccRNA	GGGAAUACUCAAGGCAAAAGCAGGAGUUUAAAUGAAUCCAAACCAGAAAAUAAUAACCAUUGGAUCGAUCUGUAUGGUA GUCGGAAUAAUUAGCCUAAUAUUGCAAAUAGUGUGAAUGGUGAUACUGUAGAUUGGUCUUGGCCAGACGGUGCUGAGC

	UCCCGUUCACCAUUGACAAGUAGUUUGUUCAAAAAACUCCUUGUUUCUACU
NA196 ccRNA-3′	GGGAAUACUCAAGGCAAAAGCAGGAGUUUAAAUGAAUCCAAACCAGAAAAUAAUAACCAUUGGAUCGAUCUGUAUGGUA<br>GUCGGAAUAAUUAGCCUAAUAUUGCAAAUAGUGUGAAUGGUGAUACUGUAGAUUGGUCUUGGCCAGACGGUGCUGAGC<br>UCCCGUUCACCAUUGACAAGUAGUUU
NA svRNA	AGUAGAAACAAGGAGUUUUUUGAAC
