# Supplementary material for: Influenza A virus transcription generates capped cRNAs that activate RIG-I
Source: bioRxiv. 2024 Dec 3:2024.11.12.623191. Originally published 2024 Nov 12. Preprint. [Version 2] doi: 10.1101/2024.11.12.623191 (PMC11601390; doi:10.1101/2024.11.12.623191)
Supplement: Supplement 3 [file media-3.pdf]

Methods

Cells

Human embryonic kidney cells (HEK293T) and Madin-Darby canine kidney cells (MDCK) were sourced from the American Type Culture Collection. Wild-type, *RIG-I* -/- and *MAVS* -/- human embryonic kidney cells stably expressing Firefly luciferase and GFP under the *IFN-β* promoter (HEK293-luc) were a generous gift from J. Rehwinkel (Oxford University) [1]. Wild-type and *RIG-I* -/- human alveolar basal epithelial cells (A549) were kindly provided by B. Ferguson (Cambridge University) [2]. Cells were cultured in Dulbecco's Modified Eagle's Medium (DMEM, Sigma-Aldrich) supplemented with 10% foetal calf serum (FCS, Gibco) and 2 mM L-glutamine (Gibco). We will refer to this medium as complete DMEM.

Plasmids

PB1 mutations described in this study, NP N319K mutation, RIG-I K270A and K861A/K858A/K851A mutations were introduced using site-directed mutagenesis using primers listed in Supplementary Table 5. The pPolI plasmids encoding A/WSN/33 genome segments were previously described in [3]; the pPolI plasmids encoding segment 5- and segment 6-based templates with internal deletions were described in [4]; the pPolI-NA d10A template was described in [5]; the pcDNA3 plasmids encoding wild-type A/WSN/33 RNP proteins in [6]; the pcDNA3 plasmid encoding PB1 D445A/D446A (PB1a) mutant in [7]; the pcDNA3 plasmid encoding PB1 R238A/R672A mutant in [8]; the pcDNA3 plasmid encoding PA with the D108A or K134A mutations in [9]; the pcDNA3 plasmid encoding TAP-tagged PB2 in [10]; the plasmid expressing firefly luciferase under *IFN-β* promoter (pIFD(−116)lucter) and pcDNA3 encoding *Renilla* luciferase in [4]; the pcDNA plasmid encoding wild-type Myc-RIG-I in [4]; MAVS-FLAG encoding plasmid and corresponding empty plasmid in [11].

Animal infections

Recombinant A/Vietnam/1203/04 (H5N1) virus was generated as described previously[12]. Recombinant virus was passaged in MDCK cells at an MOI of 0.001 before use in *in vivo* experiments. Work with highly pathogenic H5N1 virus was conducted in the Biosafety Level-3 laboratory at the LKS Faculty of Medicine, The University of Hong Kong following approved guidelines and ethics approved by the Committee on the Use of Live Animals in Teaching and Research (CULATR). BALB/c mice were infected intranasally with 10^5 PFU of A/Vietnam/1203/04 (H5N1) virus or PBS control and sacrificed 1 day post infection. Weights were recorded and virus titres in the lungs were determined using plaque assay on MDCK cells.

Generation of recombinant viruses

Wild-type and T677A mutant A/WSN/33 viruses were generated by reverse genetics using the 12-plasmid system. Briefly, eight pPolI plasmids encoding the eight IAV genome segments and four pcDNA3 plasmids encoding the IAV PB2, PB1, PA and NP proteins were transfected into 293T cells using Lipofectamine 2000 (Invitrogen) in 6 cm dishes and grown in complete DMEM. Twenty-four hours later, the cell culture medium was replaced by DMEM supplemented with 0.5% FCS and 2 mM L-glutamine. We will refer to this medium as the infection DMEM. After 48 h, cell-culture supernatants were collected, cleared by centrifugation at 1,000 rpm for 5 min, and used to infect a monolayer of MDCK cells in T75 flasks. Forty-eight hours later, cell culture supernatants were collected and cleared by centrifugation at 2,000 rpm for 10 min. The initial virus stocks were plaque purified on MDCK cells, and viruses grown from twelve individual plaques (for each wild-type and T677A virus) were subsequently assessed for DVG content using RT-PCR. Virus isolates containing the lowest amount of DVGs were selected and used for downstream experiments. Viral titres were determined by plaque assay using MDCK cells.

Virus infection

For infection experiments, cells were seeded in 24-well plates at $2*10^5$ cells/well in complete DMEM. For infection of HEK293-luc cells, wells were pre-treated with 0.5 mg/ml poly-L-lysine (Sigma-Aldrich). The next day, cells were infected with an MOI indicated for specific experiments in infection DMEM at 37 °C, 5% CO_2 for 1 h. After infection, cells were washed with PBS and incubated in infection DMEM at 37 °C, 5% CO_2. At the time periods indicated for specific experiments, supernatants were collected for virus titre quantification by plaque assay, and cells were lysed in either TRI Reagent (Sigma-Aldrich) for phenol-chloroform RNA extraction and isopropanol precipitation as described previously [13] or Laemmli buffer for western blotting.

For synchronised infections, HEK293-luc cells were seeded at $4*10^4$ cells/well in a 96-well plate pre-treated with poly-L-lysine. The next day, cells were infected with an MOI of 3 at 4 °C for 1 h. Cells were then washed with PBS and incubated in infection DMEM at 37 °C, 5% CO_2. At 3, 6, 9, 12 and 24 h time points the cell culture medium was aspirated and the firefly luciferase signal measured using DualGlo reagent (Promega) and a GloMax Microplate reader (Promega).

Serial passaging of viruses

To passage viruses A549 cells were seeded at $4*10^5$ cells per well in 12-well plates. The next day cells were infected in triplicate with wild-type or T677A mutant A/WSN/33 viruses at an

MOI of 0.01. Separate plates were used for each virus to avoid cross-contamination. Cell culture medium was collected after 48 h and cell debris were removed by pelleting at 13,000 g for 5 min. Supernatants were collected for virus quantification by plaque assay and RNA isolation using TRI Reagent (Sigma-Aldrich). Cell monolayers were lysed with Laemmli buffer for western blotting. Viral titres were measured after each passage and the titre of each new passage was used for the subsequent infection to create the next passage. A total of seven passages were performed.

Sample preparation for NGS of passaged viruses

Viral RNA collected from cell culture supernatants was isolated from Tri Reagent using phenol-chloroform extraction and isopropanol precipitation as described previously [13]. The RNA quality was determined using a Qubit RNA high sensitivity kit (Invitrogen). Eight ng of RNA was used for RT with SuperScript III (Invitrogen) and the Tuni-12 primer (5′-ACGCGTGATCAGCAAAAGCAGG). PCR amplification was done using *Pfu* DNA polymerase (Promega) and the Tuni-13 (5′- ACGCGTGATCAGTAGAAACAAGG) and Tuni-12 primers for 30 cycles. The PCR products were purified using the Monarch PCR and DNA Cleanup Kit (NEB), and the DNA concentration and purity were assessed using the Qubit dsDNA broad range kit (Invitrogen). Amplicons were pooled for each sample and next cleaned up with 0.56x Ampure XP beads to remove short DNA fragments. Next, the amplicons were sheared on Covaris LE220 sonicator to around 300 bp. Illumina sequencing adapters and barcodes were added to the sheared DNA fragments using a NEBNext Ultra II DNA Library Prep Kit for Illumina (NEB, MA) following the guide from the manufacturer. The purified DNA library products were examined on Bioanalyzer DNA High Sensitivity chips (Agilent, CA) for size distribution, quantified by Qubit fluorometer (Invitrogen, CA), and pooled at equal molar amount. The library pool was sequenced on Illumina MiSeq using a Nano 300 cycle kit as pair-end 150 nt reads. Raw sequencing reads were filtered by the MiSeq control software and only the Pass-Filter (PF) reads were used for further analysis.

Data processing pipeline of NGS for viral passaging

NGS data were analysed using the Galaxy Princeton Genomics server (galaxy.princeton.edu) [14]. First, TrimGalore! [15] was used to remove adaptor contamination, trim low-quality ends from reads with a phred quality score below 30, and filter out reads shorter than 100 nt. Read quality assessment was performed using FastQC [16]. The reference genome was based on the plasmid sequences that were used to produce viral stocks. All plasmids' sequences were confirmed using Sanger sequencing prior to read alignment. The reads were aligned to the reference genome using BWA-MEM [17]. Duplicate sequences were removed using MarkDuplicates with the following parameters: scoring strategy for non-duplicate reads - sum

of base qualities, validation strategy - lenient. Next, the BAM dataset was filtered using Filter [18] and only those reads were retained that matched the following parameters: mapping quality $\geq$ 20, 'paired', and 'properly paired'. BamLeftAlign was used to left-align reads to homogenise the positional distribution of indels. The depth of coverage was calculated using Samtools depth [19]. Variant detection was done using FreeBayes [20] with the following parameters: minimum coverage to process a site: 100; set ploidy for analysis to 1. Only variants with a frequency over 5% were scored and these are shown in Supplementary Tables 3 and 4.

Western blotting

Cells were lysed in Laemmli buffer (62.5 mM Tris-HCl pH 6.8, 10% glycerol, 2% SDS, 100 mM DTT (Invitrogen), 0.01% Bromophenol Blue) and sonicated. Proteins were separated using 8% SDS-PAGE and immobilised on a 0.45 µm nitrocellulose membrane (GE healthcare). The membranes were incubated in blocking buffer (PBS, 5% bovine serum albumin (RPI), 0.1% Tween-20 (RPI)) for 1 h, washed 3x with PBST (PBS, 0.05% Tween-20), and then incubated with the primary antibody diluted in blocking buffer for approximately 16 h at 4 °C. The following primary antibodies were used: PB1 (GTX125923, GeneTex), NP (GTX125989, Genetex), PA (GTX125932, Genetex), tubulin (MCA776, Serotec), IFIT3 B7 (sc-393512, Santa Cruz), IFIT3 (HPA059914, Sigma-Aldrich), FLAG M2 (F1804, Sigma-Aldrich), MAVS E3 (sc-166583, Santa Cruz), RIG-I (sc-376845, Santa Cruz), Myc (M4439, Sigma-Aldrich). Afterwards, the membranes were washed 3x using PBST. Binding of the primary antibodies was detected using secondary antibodies (anti-rabbit IRDye 800CW (926-32211), anti-rabbit IRDye 680RD (926-68071), anti-rat IRDye 680RD (926-68076), anti-mouse IRDye 800CW (926-32210)) sourced from LI-COR. After 1.5 h incubation with 1:10,000 dilution of secondary antibodies in blocking buffer, membranes were washed 3x with PBST and fluorescent signals were detected using an Odyssey DLx scanner (LI-COR). Signal quantification was done using the Image Studio Lite software (LI-COR).

RNP reconstitution and *IFN-β* promoter activity assay

HEK293T cells were transfected in 24-well plate format with 0.25 µg/well of pcDNA3 plasmids encoding PB2, PB1 (wild-type, T677A or D445A/D446A (PB1a) mutants), PA (wild-type, D108A or K134A mutants) and NP (wild-type or N319K mutant) and 0.25 µg/well of pPolI plasmid encoding a full-length vRNA segment or an internally truncated segment (Supplementary Table 6) under the control of the PolI promoter. NA d10A template contained a deletion of base 10 (adenosine) from the 5' vRNA terminus as described in te Velthuis, et al.

[5]. For assessment of *IFN-β* promoter activity, 0.1 µg of a plasmid expressing firefly luciferase from the *IFN-β* promoter and 0.01 µg of a plasmid expressing *Renilla* luciferase driven by the chicken β-actin promoter with a CMV enhancer were added to the transfection mix. The transfection was performed using a 1: 2.5 DNA (µg): Lipofectamine 2000 (Invitrogen) ratio. Twenty-four hours later, the complete DMEM was aspirated, cells detached with PBS, and split into fractions for RNA isolation (1/2 cell pellet), western blotting (1/4 cell pellet) or the *IFN-β* promoter activity assay (1/4 cell pellet). For *IFN-β* promoter activity assays, 25 µl of DualGlo reagent (Promega) was added to 25 µl of cells suspended in PBS. The mixture was incubated for 10 min and the firefly luciferase readings were taken using a GloMax microplate reader (Promega) or a Synergy LX plate reader (BioTek). Next, 25 µl of Stop-Glo reagent/well was added, incubated for 10 min, and the *Renilla* luciferase readings taken. Firefly luciferase values were normalised by the *Renilla* luciferase values.

Removal of capped or polyadenylated RNA

To remove capped RNAs, total RNA extracted from RNP reconstitution assays was treated sequentially with mRNA Decapping Enzyme (NEB) and XRN-1 (NEB) according to manufacturer's protocols. For removal of polyadenylated RNA, total RNA was treated with oligo $d(T)_{25}$ beads from magnetic mRNA isolation kit (NEB) according to manufacturer's protocol. The resulting RNA was purified using RNA Clean & Concentrator-5 (Zymo Research) and subsequently analysed using primer extension.

Primer extension assay

Primer extensions were performed essentially as described previously [13]. Briefly, total RNA was reverse-transcribed using Superscript III (Invitrogen) and ^{32}P-labelled primers targeting the viral RNA of interest and the cellular 5S rRNA loading control (Supplementary Table 7) in 10 µl reactions at 50 °C for 1 h. Reactions were stopped using 10 µl Formamide loading dye (90% Formamide, 10 mM EDTA, 0.25% Bromophenol Blue, 0.25% xylene cyanol FF) and denatured at 95 °C for 5 min. cDNA products were resolved on a denaturing acrylamide gel (6% acrylamide (19:1, Bio-Rad), 7 M urea, 1x Tris-borate EDTA buffer (2 mM EDTA, 89 mM boric acid, 89 mM Tris), 0.1% TEMED, 0.06% (w/v) APS) in 1x Tris-borate EDTA running buffer for 1-1.5 h at 35 W. Afterwards, the gel was dried and the radiolabelled signals visualised using phosphorimaging plates (FujiFilm) on a Typhoon FLA 9000 scanner (GE Healthcare). Signal quantification was done using Image Studio Lite software (LI-COR).

IAV RNA polymerase purification

Wild-type and mutant IAV RNA polymerases were purified using tandem-affinity purification (TAP) as described previously [13] with modifications. Briefly, 4 μg of pcDNA3 plasmids expressing wild-type or mutant PB1, PA, and PB2-TAP were transfected into HEK293T cells using 60 μg PEI in Opti-MEM (Gibco). Forty-eight hours after transfection, cells were harvested in PBS, washed once with PBS, and then lysed in 1 ml lysis buffer (50 mM HEPES pH 7.5, 200 mM NaCl, 25% glycerol (Sigma), 2% Tween-20 (RPI), 1 mM β-mercaptoethanol (Bio-Rad), and 1x EDTA-free Protease inhibitor cocktail (Roche)) at 4 °C for 1 h. Next, the lysed cells were sonicated to disrupt DNA and RNA molecules, and centrifuged at 17000 x *g* for 5 min at 4 °C. The cleared lysates were bound to 50 μl IgG Sepharose beads 6 Flow (GE Healthcare) that were pre-washed 3x in binding buffer (50 mM HEPES pH 8, 200 mM NaCl, 25% glycerol, 2% Tween-20). The binding was performed under constant rotation at 4 °C. After 2 h, the beads were washed 3x with binding buffer and once with cleavage buffer (50 mM HEPES pH 7.5, 200 mM NaCl, 25% glycerol, 0.5% Tween, 1 mM DTT). Finally, the TAP-tag was removed using Tobacco etch virus (TEV) protease (Invitrogen, #12575-015) in 250 μl of cleavage buffer. Cleavage was performed at 4 °C for approximately 16 h. The beads were separated from the cleaved RNA polymerase by centrifugation at 500 *x g* for 1 min. The partially purified RNA polymerase was analysed through SDS-PAGE and silver staining using a SilverXpress kit (Invitrogen).

Activity assays with the purified IAV polymerase

To measure the ability of the IAV RNA polymerase to extend a capped primer, first a synthetic 11-nt long RNA with 5' diphosphate (Chemgenes; Supplementary Table 8) was capped with a radiolabelled cap-1 structure in 20 μl reactions containing 1 μM RNA, 0.25 μM [α-^{32}P]GTP (3000 Ci/mmole, Perking-Elmer), 0.8 mM S-adenosylmethionine, 0.5 U/μl *Vaccinia virus* capping enzyme (NEB), and 2.5 U/μl 2'-O-methyltransferase (NEB) at 37 °C for 1 h. Next, the capped RNA was purified using an oligonucleotide cleanup kit (Zymo Research) and eluted in 30 μl water. To test the transcriptional activity of the IAV RNA polymerase, we set up 4-μl reactions containing 5 mM $MgCl_2$, 1 mM DTT, 2 U/μl RNase inhibitor (APExBIO), 0.5 μM of RNA template (Supplementary Table 8), 500 μM ATP, 500 μM CTP, 500 μM GTP, ~1500 cpm capped RNA primer, 12.5% glycerol, 1% Tween-20, 100 mM NaCl, 25 mM HEPES pH 8, 50 μM baloxavir (AstaTech), and 5 ng/μl RNA polymerase. Reactions were incubated at 30 °C for 1 h and analysed with 12% denaturing PAGE and autoradiography.

For the RNase H analysis, transcription reactions were performed as described above and stopped by boiling at 95 °C for 2 min. Next, 1 μM oligo $d(T)_{20}$ and 2.5 U RNase H (NEB) were added and the samples incubated at 37 °C for 30 min. The RNase-treated reactions were analysed with 12% denaturing PAGE and autoradiography.

For the primer extension reactions with $VNdT_{20}$ primer (Supplementary Table 7), the RNA polymerase reactions were performed with a non-radiolabelled capped primer (Supplementary Table 8). This primer was produced by substituting the [α-^{32}P]GTP with 500 μM GTP in the capping reaction. The transcription reactions were stopped after 2 h by boiling at 95 °C for 2 min. Next, the RNA was extracted using Tri-Reagent and isopropanol precipitation. Finally, the RNA was reverse-transcribed using Superscript III (Invitrogen) and a ^{32}P-labelled $VNdT_{20}$ primer (Supplementary Table 7) in 10 μl reactions at 42 °C for 1 h. The primer extension reactions were analysed with 12% denaturing PAGE and autoradiography.

Promoter binding assays

Recombinant polymerases were purified using a TAP-tag on the C-terminus of the PB2 subunit. Briefly, HEK293T cells were transfected with 4 μg of pcDNA3 plasmids encoding PB1 (wild-type, T677A or R238A/R672A), PB2 (TAP-tagged or untagged) and PA in a 10 cm dish using Polyethylenimine (PEI, Sigma) transfection reagent at a 1: 3 (DNA (μg): PEI (μl)) ratio. Forty-eight hours after transfection cells were lysed with cold lysis buffer (50 mM HEPES (pH 8.0), 200 mM NaCl, 25% glycerol, 0.5% NP-40, 0.007% β-mercaptoethanol, 1x cOmplete protease inhibitor cocktail (Roche)) on ice. Debris were removed by centrifugation at 13,000 g and 4 °C for 5 min. Next, the NaCl concentration of the supernatant was returned to 150 mM. The supernatant was incubated with 50 μl of IgG Sepharose beads (GE Healthcare) (pre-washed 5x with binding buffer (10 mM HEPES (pH 8.0), 150 mM NaCl, 10% glycerol, 0.1% NP-40)) at 4 °C for 2 h. Next, the IgG Sepharose beads were washed 3x with binding buffer and stored at -70 °C.

To test the ability of the RNA polymerase to bind the viral promoter, 10 μM of 3′ vRNA terminus (3′-UCGUUUCGUCCUCAAA) labelled with ATTO647N [21] and 10 μM of unlabelled 5′ vRNA terminus (5′-AGUAGAAACAAGGAGUUU) were first annealed using a step-wise protocol (90 °C for 1 min, a ramp down to 25 °C over 1 h, and finally cooling at 4 °C). Polymerase-bound beads were washed with promoter binding buffer (50 mM Tris-HCl (pH 8.0), 500 mM NaCl, 10 mM $MgCl_2$, 0.1 mg/ml bovine serum albumin, 5% glycerol and 1 mM DTT). Next, beads were incubated with 1 μl of annealed promoter in promoter binding buffer supplemented with 1U/ul RNaseOUT™ Recombinant Ribonuclease Inhibitor (ThermoFisher) for 30 min at 30 °C and shaking at 1,2000 rpm. Samples were transferred to ice and crosslinked for 10 min using 254 nm UV light in a Stratalinker UV 1800 (Stratagene). Crosslinked samples were resuspended in Laemmli buffer, boiled at 95 °C for 5 min, and resolved using 8% SDS-PAGE. The ATTO647N signal was visualised at 700 nm using Odyssey DLx scanner (LI-COR). The same gel was subsequently used for western blotting.

For assays measuring full-length vRNA binding to NA, transfections for the TAP-tag purification included 4 μg of a pPolI plasmid encoding a full-length NA segment. The polymerase-vRNA complex was bound to IgG Sepharose beads as described above. RNA and protein samples were collected from the total lysate and the bead-bound fractions, and these samples were analysed using primer extension with NA 1280 and 5S 100 primers (Supplementary Table 7).

RT-PCR for detection of viral genome segments and DVGs

Reverse transcription reactions were done using total RNA isolated from infected cells or virus-containing supernatants, Tuni-12 (5′- ACGCGTGATCAGCAAAAGCAGG) and Tuni-13 (5′-ACGCGTGATCAGTAGAAACAAGG) primers [22], and SuperScript III (Invitrogen) at 50 °C for 1 h. PCR reactions were done using Tuni-12 and Tuni-13 primers and *Pfu* DNA polymerase (Promega). PCR products were resolved on a 1.5% 0.5x TAE agarose gel.

RT-PCR for detection of mvRNA

RNA isolated from infection samples was fractionated by length using an RNA Clean & Concentrator kit (Zymo Research). The small (<200 nt) RNA fraction was used for reverse transcription with Lv3aa and Lv3ga primers (Supplementary Table 9) and SuperScript III (Invitrogen) at 37 °C for 30 min as described previously [4]. Second strand synthesis was done using the Lv5 primer (Supplementary Table 9) and *Pfu* DNA polymerase (Promega) at 95 °C for 2 min, 47 °C for 10 min and 72 °C for 3 min. Next, samples were treated with Exonuclease VII (NEB) at 37 °C for 30 min and 95 °C for 10 min. PCR was done with P7 and P5_IRdye800 (conjugated to an IRdye800 fluorophore) (Supplementary Table 9) primers and *Pfu* DNA polymerase. Thermocycling conditions included: i) initial denaturation at 95 °C for 2 min; ii) 24-28 cycles of denaturation at 95 °C for 30 s, annealing at 59 °C for 30 s and extension at 72 °C for 30 s; iii) final extension at 72 °C for 5 min. RT-PCR for 5S rRNA was done as described above (without second strand synthesis) using the 5S 100 primer for reverse transcription and 5S_Fw and 5S100_Rev_ATTO647N (conjugated to an ATTO647N fluorophore) primers for PCR. (Supplementary Table 9). Five μl of 6x Orange G loading dye (6x concentration: 10 mM Tris (pH 7.5), 50% glycerol, 1 mM EDTA, Orange G) was added and samples were run on a 6% acrylamide TBE gel. RT-PCR products were visualised using 700 nm (for 5S rRNA) or 800 nm (for mvRNA) wavelengths on an Odyssey DLx scanner (LI-COR). Gels were subsequently stained with SYBR Gold (Invitrogen) to visualise the ladder using a G:BOX XX6 gel imager (Syngene).

Immunoprecipitation of Myc-tagged RIG-I

For immunoprecipitation assays with full-length IAV segments, HEK293T cells were transfected with 3 µg of each of the following plasmids: pcDNA3-NP, pcDNA3-PB2, pcDNA3-PA, pcDNA3-PB1 (wild-type or T677A mutant), pcDNA-Myc-RIG-I (wild-type or K861A/K858A/K851A mutant) and pPolI plasmid encoding a full-length NA or NP segment. The transfection was done using 1: 2.5 DNA (µg): Lipofectamine 2000 (Invitrogen) ratio in 10 cm dishes. Forty-eight hours later, cells were detached with 10 ml of cold PBS, pelleted at 1,000 rpm for 5 min at 4 °C and lysed in 600 µl of cold lysis buffer (50 mM Tris-HCl (pH 8.0), 5% glycerol, 0.5% NP-40, 200 mM NaCl, 1 mM EDTA, 1 mM DTT, cOmplete protease inhibitor cocktail (Roche)) at 4 °C for 1 h. The cell debris were removed by pelleting at 13,000 rpm, 4 °C for 5 min and 20% of the sample was taken for 'total' RNA and protein analyses. The remaining lysate was incubated with 1.5 µl of anti-Myc antibody (#M4439, Sigma-Aldrich) at 4 °C for 2 h under rotation. Twenty-five µl of Protein G Mag Sepharose Xtra beads (GE Healthcare) per condition was washed 3x with 1 ml of wash buffer (10 mM Tris-HCl (pH 8.0), 150 mM NaCl, 0.1% NP-40, 1 mM EDTA). Then lysates were added to the beads and incubated overnight at 4 °C under rotation. Next, beads were washed 3x with wash buffer at 4 °C for 5 min under rotation. Finally, two thirds of the bead suspension was used for RNA extraction and one third for protein analysis. RNA samples were analysed by primer extension with NA160 and NA1280 or NP 149- and NP149+ primers (Supplementary Table 7).

For immunoprecipitations with the NA196 template, 10 cm dishes with HEK293T cells were transfected with 4 µg of each of the following plasmids: pcDNA3-NP, pcDNA3-PB2, pcDNA3-PA, pcDNA3-PB1 (wild-type or T677A mutant), pcDNA-Myc-RIG-I (wild-type, K861A/K858A/K851A, K270A mutant) or empty pcDNA3 plasmid and pPolI plasmid encoding the NA196 template. Transfections were done using a 1: 5 (DNA (µg): PEI (µl)) ratio. Forty-eight hours after transfection, cells were washed with cold PBS and lysed with 600 µl of cold lysis buffer (10 mM Tris-HCl pH 7.5, 150 mM NaCl, 0.5% NP-40, 1x cOmplete protease inhibitor cocktail (Roche)) on ice for 1 h. Cell debris were removed through centrifugation at 17,000 rpm, 4 °C for 20 min. Next, 900 µl of dilution buffer (10 mM Tris-HCl (pH 7.5), 150 mM NaCl, cOmplete protease inhibitor cocktail (Roche)) was added to the supernatants. Ten percent of the supernatants was saved for 'total' RNA and protein analyses. Twenty-five µl of ChromoTek Myc-Trap® Agarose beads (proteintech) (pre-wasehed 3x with wash buffer (10 mM Tris-HCl (pH 7.5), 200 mM NaCl, 0.05% NP-40)) were added to the remaining supernatant and incubated at 4 °C for 1 h under rotation. Next, the beads were washed 3x with wash buffer and two-thirds of the bead suspension was used for RNA extraction and one third for protein analysis. RNA samples were analysed by primer extension with the NA 5' or NA-2 primer (Supplementary Table 7).

TSO-based RT-PCR and gel electrophoresis

For optimisation of the TSO-based RT-PCR assay, 0.625 ng of *in vitro* transcribed vRNA, cRNA, ccRNA, ccRNA-3', mRNA or combinations thereof were mixed with 10 ng of total HEK293T RNA and subsequently used as input for the reactions. For the TSO-based RT-PCR analysis of tissue culture infection samples, RNA was extracted from A549 cells infected with an MOI of 0.3 of wild-type or T677A A/WSN/33 virus for 24 h. For analysis of mouse infection samples, RNA was isolated from mouse lungs 1 day after infection with 10^5 PFU of A/Vietnam/1203/04 (H5N1). Next, RNA was extracted, and 2 µg of total RNA was either treated or mock-treated with oligo $d(T)_{25}$ beads from the magnetic mRNA isolation kit (NEB) according to manufacturer's instructions. RNA was subsequently purified using an RNA Clean & Concentrator-5 kit (Zymo Research). RNA from TSO-based RT was done with equal volumes (containing 100-200 ng) of RNA. To this end, RNA was first denatured in the presence of dNTPs (1mM final concentration) and either the Tuni-13, the Tuni-13 LNA3, or the oligo $d(T)_{20}$ primer (1 µM final concentration, Supplementary Table 10) in 3 µl volume at 70 °C for 5 min. (Note that Tuni-13 LNA3 primer has slightly better specificity in comparison to the Tuni-13 primer. Additionally, we recommend to always include the controls with and without poly(A) depletion and reverse transcription for both ccRNA and mRNA to control for inherently unspecific nature of reverse transcriptases in this method.) Denatured RNAs were immediately placed on ice for 5 min. Next, 2 µl of enzyme mix containing template switching RT buffer (NEB), template switching RT enzyme mix (NEB), and the TSO (3.75 µM final concentration, Supplementary Table 10) were added to the RNA mix and incubated at 42 °C for 90 min. Reactions were terminated by incubation at 85 °C for 5 min and subsequently cooled to 4 °C. The unused primers were digested with Thermolabile Exonuclease I (NEB) and reactions were diluted 2x with water. One µl of diluted RT reaction was used for PCR amplification with Q5 High-Fidelity DNA polymerase (NEB) using TSO-specific forward primer and a gene-specific reverse primer (Supplementary Table 10). Thermocycling conditions included: i) initial denaturation at 98 °C for 30 s; ii) 25-29 cycles of denaturation at 98 °C for 10 s, annealing at 50 °C for 15 s and extension at 72 °C for 10 s; iii) final extension at 72 °C for 2 min. Note that due to abundance of mRNA in infection, lower number of PCR amplification cycles was used for mRNA than for cRNA/ccRNA to avoid oversaturation. The PCR products were resolved on 8-10% TBE acrylamide gel and visualised using SYBR Gold (Invitrogen) and G:BOX XX6 gel imager (Syngene).

To determine the efficiency of the mRNA depletion with oligo $d(T)_{25}$ beads, RNA from TSO-based RT reactions with either the Tuni-13 LNA3, oligo $d(T)_{20}$ or 18S rRNA reverse primer (Supplementary Table 10) was treated with Thermolabile Exonuclease I (NEB) and used for qPCR. qPCR reactions were done using NA-specific or 18S rRNA-specific primers (Supplementary Table 10) and qRT-PCR Brilliant III SYBR Master Mix with ROX (Agilent).

TSO-based RT-PCR and NGS library preparation

A549 cells were seeded in a 6-well plate in complete DMEM a day before the infection. Media were changed to IAV growth medium (Opti-MEM, 0.04% BSA fraction V, 100 µg/ml $CaCl_2$, 0.01% FBS) before cells were infected at an MOI of 1 with either A/WSN/33 wild-type or T677A mutant virus. RNA was harvested from infected cells 16 h post-infection and purified using the Monarch Total RNA Miniprep Kit (NEB).

As controls for PCR chimeras and template switching during reverse transcription, RNA samples were spiked (1%) with RNA extracted from a 16 h transfection of pPolI plasmids encoding the eight wild-type A/WSN/33 virus segments with synonymous mutations at the 5' and 3' termini [23]. Mixed RNA samples were reverse transcribed with the Template Switching RT Enzyme Mix (NEB) according to the manufacturer's protocol and subsequently amplified and appended with the partial Illumina i5 and i7 adapter sequences using Q5 Hot Start High-Fidelity 2x Master Mix (NEB). In brief, 4 µl of sample was annealed with the 3' cRNA primer (Supplementary Table 11) [24] and then reverse transcribed with TSO 2 (Supplementary Table 11) containing a 5' biotin modification to prevent spurious additional concatemerization similar as described by Zajac, et al. [25] and Picelli, et al. [26]. The 5' terminal ends were amplified with a TSO-specific circularization primer and a primer mix of the Hoffmann 3' - Ba/Bm primers for all eight segments [24] (Supplementary Table 11) with an extension time of 1 min, 30 s and a final annealing temperature of 66 °C. Next, samples were cleaned using beads at 1.5x v/v (beads: sample) using ProNex Size-Selective Purification System (Promega). Gibson assembly was performed for 1 h using NEBuilder HiFi DNA Assembly Master Mix (NEB) with 10 ng of product. Assembly reactions were subsequently digested at 37 °C for 1 h using Exonuclease V (NEB), supplemented with ATP (NEB), to remove remaining linear amplicons. After an additional cleanup with 1.5x v/v beads, samples underwent segment 8-specific amplifications using NSrnd1UP and NSrnd1DWN primers (65 °C annealing temperature, 30 s extension time, 28 cycles) using Q5 Hot Start High-Fidelity 2x Master Mix (NEB) before Illumina partial adapters (NSrnd2UP and NSrnd2DWN) were appended (65 °C annealing temperature, 15 s extension time, 10 cycles) (Supplementary Table 11). Samples were pooled and cleaned with 2x v/v beads prior to adding Nextera indices (62 °C annealing temperature, 20 s extension time, 7 cycles). Samples were resolved on a 1% TAE agarose gel, extracted, and column and bead purified with 3x v/v beads. Finally, the sample purity and concentration were assessed via nanodrop and Qubit, respectively.

Computation analysis of TSO-based RT-PCR sequencing

Samples were sequenced on a MiSeq using 2x250 chemistry. All reads were processed with Trimmomatic to remove low-scoring positions, followed by mapping with STAR; excluding

splice mapping; filtering out mismatches of greater than 3, with deletion and gap open scores of 6 and enforcing end-to-end mapping; and using a single thread to maintain read order from the FASTQ files. STAR mapping was used to infer to which segment each read should align, but not to determine the TSO location or cap identity. We used perfect matching to identify the cap sequence, and then enforced perfect matching to each segment on either side of the circularized template to determine the TSO location. The appropriate distance from the TSO was next calculated. This was a conservative approach given that perfect matches are required. In addition, the anticipated distance from the read initiation to the 5' (or 3') end of the cRNA/ccRNA was required to be within 3 nt of the expected end, again enforcing appropriate matches to the expected template. In addition, it required that the distance to the TSO from the 5' end of the template would be at least 0, as internal "deletions" could represent TSO priming at the 5' end rather than be products of true template switching.

We could not use our spike RNA to control for template switching as the set parameters left few enough reads to produce sufficient data. Instead, we rely on the apparent rate of inter-segmental chimeras, which could result from template switching or linear concatemer formation, to estimate the frequency at which these artifacts contaminate our data. Of the ~12,000 reads that meet our requirements across all six samples, only eight meet these rejection criteria, far below the frequency at which we observe ccRNA (and cRNA), indicating that these processes are unlikely to explain our data. The full pipeline is available at https://github.com/a5russell/ccRNA_miSeq.

In vitro transcription and RNA transfection

DNA templates for *in vitro* transcription were prepared by PCR using the primers listed in Supplementary Table 12, Q5 High-Fidelity DNA polymerase (NEB), and the pPolI-NA196 plasmid as a template. The DNA template for the svRNA *in vitro* transcription was prepared by annealing svRNA forward and reverse oligos (Supplementary Table 12) using a stepwise protocol (90 °C for 1 min, ramping down to 25 °C over 1h, and holding at 4 °C). *In vitro* transcriptions were done using MEGAshortscript™ T7 Transcription Kit (ThermoFisher) according to manufacturer's protocol. The products were resolved on a denaturing acrylamide gel (6% or 15% Acrylamide (19:1, Bio-Rad) (for longer RNA or svRNA respectively), 7M urea, 1x Tris-borate EDTA buffer (2 mM EDTA, 89 mM boric acid, 89 mM Tris), 0.1% TEMED, 0.06% (w/v) APS) in 1x Tris-borate EDTA running buffer. The products were gel-purified by freezing gel slices on dry ice, crushing the slices, and incubating the slices with elution buffer (20 mM Tris-HCl pH 7.5, 0.25 M sodium acetate, 1 mM EDTA, and 0.25% SDS) (for longer RNAs) or water (for svRNA) at RT overnight, under constant shaking. The RNA was subsequently purified using RNA Clean & Concentrator-5 (Zymo Research), increasing ethanol concentration during column binding to 66% for the svRNA purification. Part of the cRNA

sample was dephosphorylated using Antarctic phosphatase (NEB) according to manufacturer's protocol. The ccRNA, mRNA and ccRNA-3′ transcripts were capped and methylated using Vaccinia Capping Enzyme (NEB) and cap 2´-O-methyltransferase (NEB) according to manufacturer's protocol. Afterwards, ccRNA, mRNA and ccRNA-3′ were treated sequentially with Antarctic phosphatase (NEB), PNK (NEB) and XRN-1 (NEB) to remove uncapped RNA according to manufacturer's protocols. The RNA was purified using RNA Clean & Concentrator-5 (Zymo Research) in between treatments. The quality of the final RNA preparations was assessed using denaturing acrylamide gel electrophoresis. Sequences of expected *in vitro* transcription products are listed in Supplementary Table 13.

The *in vitro* transcribed RNAs were transfected alone or in combination with svRNA or pPolI plasmids encoding segment 6- or segment 5 -based vRNAs into HEK293-luc or HEK293-luc *RIG-I* -/- cells together with *Renilla* luciferase-encoding plasmid and RNAse inhibitor (APExBIO) using Lipofectamine 2000 (Invitrogen) reagent. In all transfections, the *in vitro* transcribed RNA was diluted in total HEK293T RNA, which was also used as a negative control. The MAVS-FLAG encoding plasmid was used as a positive control as described previously [11]. Firefly luciferase and *Renilla* luciferase readings were taken using Dual-Glo® Luciferase Assay System (Promega) 20 h post-transfection.

RIG-I activation assay

RIG-I was purified as described previously [27]. For ATPase assays, 0.5 µM RIG-I was incubated with 0.1 µM [γ-^{32}P]ATP and 10 ng of template RNA. Activity assays were performed in a buffer containing 50 mM HEPES pH 8.0, 150 mM NaCl, 2 mM $MgCl_2$, and 5 mM DTT, and quenched using 1M formic acid/50 mM EDTA. [γ-^{32}P]ATP and $^{32}P_i$ were resolved using glass-backed PEI-cellulose TLC plates (Sigma-Aldrich) in 0.4 M KH_2PO_4 (pH 3.4). After wrapping the TLC plates in plastic foil, the radioactive signals were detected using BAS-MS phosphorimaging plates (FujiFilm) through a 1 h exposure and visualised using a Typhoon FLA 9000 scanner (GE Healthcare). Densitometry analysis was performed using ImageJ.

Statistical analysis

In all figures, error bars indicate standard deviation with the number of biological repeats indicated in figures or figure legends. Statistical analysis was done using GraphPad Prism Software. Details on the tests used are indicated in each figure legend.

References for methods

1 Hertzog, J. *et al.* Infection with a Brazilian isolate of Zika virus generates RIG-I stimulatory RNA and the viral NS5 protein blocks type I IFN induction and signaling. *Eur J Immunol* **48**, 1120-1136 (2018). https://doi.org/10.1002/eji.201847483

2 Teague, H. C. *et al.* LUBAC is required for RIG-I sensing of RNA viruses. *Cell Death Differ* **31**, 28-39 (2024). https://doi.org/10.1038/s41418-023-01233-x

3 Fodor, E. *et al.* Rescue of influenza A virus from recombinant DNA. *J Virol* **73**, 9679-9682 (1999). https://doi.org/10.1128/JVI.73.11.9679-9682.1999

4 te Velthuis, A. J. W. *et al.* in *Nature Microbiology* Vol. 3 1234-1242 (2018).

5 te Velthuis, A. J., Turrell, L., Vreede, F. T. & Fodor, E. Uncoupling of influenza A virus transcription and replication through mutation of the unpaired adenosine in the viral RNA promoter. *J Virol* **87**, 10381-10384 (2013). https://doi.org/10.1128/JVI.00636-13

6 Fodor, E. *et al.* A single amino acid mutation in the PA subunit of the influenza virus RNA polymerase inhibits endonucleolytic cleavage of capped RNAs. *J Virol* **76**, 8989-9001 (2002). https://doi.org/10.1128/jvi.76.18.8989-9001.2002

7 Vreede, F. T., Jung, T. E. & Brownlee, G. G. Model suggesting that replication of influenza virus is regulated by stabilization of replicative intermediates. *J Virol* **78**, 9568-9572 (2004). https://doi.org/10.1128/JVI.78.17.9568-9572.2004

8 Te Velthuis, A. J., Robb, N. C., Kapanidis, A. N. & Fodor, E. The role of the priming loop in influenza A virus RNA synthesis. *Nat Microbiol* **1**, 16029 (2016). https://doi.org/10.1038/nmicrobiol.2016.29

9 Hara, K., Schmidt, F. I., Crow, M. & Brownlee, G. G. in *Journal of Virology* Vol. 80 7789-7798 (American Society for Microbiology, 2006).

10 Deng, T., Sharps, J., Fodor, E. & Brownlee, G. G. In vitro assembly of PB2 with a PB1-PA dimer supports a new model of assembly of influenza A virus polymerase subunits into a functional trimeric complex. *J Virol* **79**, 8669-8674 (2005). https://doi.org/10.1128/JVI.79.13.8669-8674.2005

11 French, H. *et al.* Transient RNA structures cause aberrant influenza virus replication and innate immune activation. *Sci Adv* **8**, eabp8655 (2022). https://doi.org/10.1126/sciadv.abp8655

12 Cheung, P. P. *et al.* Generation and characterization of influenza A viruses with altered polymerase fidelity. *Nat Commun* **5**, 4794 (2014). https://doi.org/https://doi.org/10.1038/ncomms5794

13 Aartjan J. W. te Velthuis, J. S. L., Wendy S. Barclay. in *Influenza Virus: Methods and Protocols Methods in Molecular Biology* (ed Yohei Yamauchi) 343-374 (Springer Nature, 2018).

14 Afgan, E. *et al.* The Galaxy platform for accessible, reproducible and collaborative biomedical analyses: 2018 update. *Nucleic Acids Res* **46**, W537-W544 (2018). https://doi.org/10.1093/nar/gky379

15 Krueger, F. <https://www.bioinformatics.babraham.ac.uk/projects/trim_galore/> (n.d.).

16 Andrews, S. *Fast QC A Quality Control tool for High Throughput Sequence Data.*, <http://www.bioinformatics.babraham.ac.uk/projects/fastqc/> (n.d.).

17 Li, H. & Durbin, R. Fast and accurate short read alignment with Burrows-Wheeler transform. *Bioinformatics* **25**, 1754-1760 (2009). https://doi.org/10.1093/bioinformatics/btp324

18 Barnett, D. W., Garrison, E. K., Quinlan, A. R., Stromberg, M. P. & Marth, G. T. BamTools: a C++ API and toolkit for analyzing and managing BAM files. *Bioinformatics* **27**, 1691-1692 (2011). https://doi.org/10.1093/bioinformatics/btr174
19 Li, H. *et al.* The Sequence Alignment/Map format and SAMtools. *Bioinformatics* **25**, 2078-2079 (2009). https://doi.org/10.1093/bioinformatics/btp352
20 Garrison E., G., M. *Haplotype-based variant detection from short-read sequencing*, <https://arxiv.org/abs/1207.3907> (2012).
21 Robb, N. C. *et al.* in *Nucleic Acids Research* Vol. 44 10304-10315 (2016).
22 Xue, J., Chambers, B. S., Hensley, S. E. & Lopez, C. B. Propagation and Characterization of Influenza Virus Stocks That Lack High Levels of Defective Viral Genomes and Hemagglutinin Mutations. *Front Microbiol* **7**, 326 (2016). https://doi.org/10.3389/fmicb.2016.00326
23 Russell, A. B., Trapnell, C. & Bloom, J. D. Extreme heterogeneity of influenza virus infection in single cells. *Elife* **7** (2018). https://doi.org/https://doi.org/10.7554/eLife.32303
24 Hoffmann, E., Stech, J., Guan, Y., Webster, R. G. & Perez, D. R. Universal primer set for the full-length amplification of all influenza A viruses. *Arch Virol* **146**, 2275-2289 (2001). https://doi.org/10.1007/s007050170002
25 Zajac, P., Islam, S., Hochgerner, H., Lonnerberg, P. & Linnarsson, S. Base preferences in non-templated nucleotide incorporation by MMLV-derived reverse transcriptases. *PLoS One* **8**, e85270 (2013). https://doi.org/10.1371/journal.pone.0085270
26 Picelli, S. *et al.* Full-length RNA-seq from single cells using Smart-seq2. *Nat Protoc* **9**, 171-181 (2014). https://doi.org/10.1038/nprot.2014.006
27 Devarkar, S. C., Schweibenz, B., Wang, C., Marcotrigiano, J. & Patel, S. S. RIG-I Uses an ATPase-Powered Translocation-Throttling Mechanism for Kinetic Proofreading of RNAs and Oligomerization. *Mol Cell* **72**, 355-368 e354 (2018). https://doi.org/10.1016/j.molcel.2018.08.021
