# Supplementary material for: Influenza A virus transcription generates capped cRNAs that activate RIG-I
Source: bioRxiv. 2024 Nov 12:2024.11.12.623191. Preprint. [Version 1] doi: 10.1101/2024.11.12.623191 (PMC11601390; doi:10.1101/2024.11.12.623191)
Supplement: Supplementary file 1 [file media-1.pdf]

## Supplementary Tables

# Influenza A virus transcription generates capped cRNAs that activate RIG-I

Elizaveta Elshina[1,2], Emmanuelle Pitre[1,2], Marisa Mendes[3], Brandon Schweibenz[4], Hollie French[2,#], Ji Woo Park[1], Wei Wang[5], Joseph Marcotrigiano[4], Alistair B. Russell[3], Aartjan J.W. te Velthuis[1,*]

**Supplementary Table 1. Amino acid variation at position 677 of IAV PB1 (analysis done using www.fludb.org database).**

| RESIDUE 677 | NO. OF STRAINS (% CONSERVATION) |
|---|---|
| T | 45,293 (99.98) |
| S | 3 (0.01) |
| M | 1 (0.00) |
| A | 1 (0.00) |
| P | 3 (0.01) |

**Supplementary Table 2. Variants identified in the stock and passages of the wild-type A/WSN/33 virus.**

| SEGMENT | POSITION | TYPE | REFERENCE | ALLELE | AMINO ACID/ORF CHANGE | VARIANT FREQUENCY (%) | | | |
|---|---|---|---|---|---|---|---|---|---|
| | | | | | | **stock** | **P7 (1)** | **P7 (2)** | **P7 (3)** |
| **3 (PA)** | 1711 | snp | T | C | silent | 99 | 99 | 99 | 99 |
| **6 (NA)** | 66 | snp | T | C | V16A | 82 | | | |
| **5 (NP)** | 143 | snp | T | C | I33T | 74 | 36 | 35 | 27 |
| **6 (NA)** | 1368 | snp | C | G | T450S | | 100 | 100 | 100 |
| **6 (NA)** | 1103 | snp | A | G | N362D | | 82 | 77 | 82 |
| **6 (NA)** | 1325 | snp | A | G | T436A | | 78 | 78 | 71 |
| **6 (NA)** | 1330 | snp | A | G | silent | | 77 | 78 | 71 |
| **3 (PA)** | 244 | del | CACAGATTTGAAATA | CA | frameshift (81 aa product) | | 30 | | |
| **3 (PA)** | 166 | del | TATTCA | TA | stop (47 aa product) | | 26 | | |
| **3 (PA)** | 51 | del | CAATC | CATC | frameshift (11 aa product) | | 26 | | |
| **3 (PA)** | 1943 | del | TATTGGCAAAGTCGGTATT CAACAGCTTGTATGCA | TA | del 641-651 | | 15 | | |
| **1 (PB2)** | 2300 | snp | T | C | I760T | | 14 | | |
| **2 (PB1)** | 1882 | del | TACCAGGGGCGTTTATGCA | TA | stop (619 aa product) | | | 14 | |

**Supplementary Table 3. Variants identified in the stock and passages of the T677A A/WSN/33 virus.**

| SEGMENT | POSITION | TYPE | REFERENCE | ALLELE | AMINO ACID/ORF CHANGE | VARIANT FREQUENCY (%) | | | | | | |
|---|---|---|---|---|---|---|---|---|---|---|---|---|
| | | | | | | **stock** | **P5 (1)** | **P7 (1)** | **P3 (2)** | **P4 (2)** | **P4 (3)** | **P5 (3)** |
| **2 (PB1)** | 2053 | snp | A | G | T677A | 100 | 99 | 100 | 13 | 9 | 14 | 8 |
| **5 (NP)** | 1002 | snp | T | G | N319K | | 67 | 80 | 24 | 47 | 22 | 45 |
| **2 (PB1)** | 1650 | snp | A | T | silent | | 34 | 73 | | | | |
| **2 (PB1)** | 776 | snp | T | G | F251C | | 33 | 71 | | | | |
| **5 (NP)** | 1175 | snp | G | A | S377N | | 19 | 14 | | | | |
| **4 (HA)** | 1583 | snp | T | A | D517E | | 18 | 21 | | | | |
| **1 (PB2)** | 123 | snp | G | A | silent | | 18 | 21 | | | | |
| **5 (NP)** | 37 | snp | A | G | non-coding region | | 16 | 12 | | | | |
| **2 (PB1)** | 516 | snp | A | G | I164M | | 14 | 11 | | | | |
| **4 (HA)** | 1136 | snp | T | C | silent | | 10 | 7 | | | | |
| **4 (HA)** | 1160 | snp | C | A | silent | | | | 58 | 60 | | |

| | | | | | | | | | |
|---|---|---|---|---|---|---|---|---|---|
| **8 (NS)** | 553 | snp | A | T | N176I (NS1) M19L (NEP) | 23 | 22 | 40 | 35 |
| **5 (NP)** | 1450 | snp | G | A | E469K | 15 | 16 | 13 | 12 |
| **2 (PB1)** | 2226 | snp | G | A | silent | 15 | 10 | 11 | 12 |
| **4 (HA)** | 116 | snp | C | T | silent | 15 | 12 | 14 | 13 |
| **1 (PB2)** | 2226 | snp | G | A | silent | 11 | 9 | 9 | 12 |
| **4 (HA)** | 1660 | snp | T | A | stop (542 aa product) | 10 | 11 | 10 | 11 |
| **5 (NP)** | 711 | snp | G | A | M222I | | | 9 | 11 |
| **3 (PA)** | 243 | del | GCAC | GC | frameshift (75 aa product) | | | 9 | 24 |
| **3 (PA)** | 115 | del | GAAACAAACA<br>AAT | GAAACAA<br>AT | frameshift (59 aa product) | | | 7 | 22 |

**Supplementary Table 4. Primers used for site-directed mutagenesis.**

| PRIMER NAME | MUTATION | PRIMER SEQUENCE (5′- 3′) |
|---|---|---|
| PB1 R670A Fw | PB1 R670A | CTCCTGGATCCCCAAAGCAAATCGATCCATCTTG |
| PB1 R670A Rev | PB1 R670A | CAAGATGGATCGATTTGCTTTGGGGATCCAGGAG |
| PB1 N671A Fw | PB1 N671A | GGATCCCCAAAAGAGCTCGATCCATCTTGAATACAAGC |
| PB1 N671A Rev | PB1 N671A | GCTTGTATTCAAGATGGATCGAGCTCTTTTGGGGATCC |
| PB1 S673A Fw | PB1 S673A | GGATCCCCAAAAGAAATCGAGCTATCTTGAATACAAGCC |
| PB1 S673A Rev | PB1 S673A | GGCTTGTATTCAAGATAGCTCGATTTCTTTTGGGGATCC |
| PB1 N676A Fw | PB1 N676A | GGATCCCCAAAAGAGCTCGATCCATCTTGAATAC |
| PB1 N676A Rev | PB1 N676A | GTATTCAAGATGGATCGAGCTCTTTTGGGGATCC |
| PB1 T677A Fw | PB1 T677A | CATCTTGAATGCAAGCCAAAG |
| PB1 T677A Rev | PB1 T677A | GATCGATTTCTTTTGGGG |
| PB1 S678A Fw | PB1 S678A | CGATCCATCTTGAATACAGCTCAAAGAGGAATACTTG |
| PB1 S678A Rev | PB1 S678A | CAAGTATTCCTCTTTGAGCTGTATTCAAGATGGATCG |
| NP N319K Fw | NP N319K | CAGCCTAATCAGACCAAAGGAGAATCCAGCACACAAG |
| NP N319K Rev | NP N319K | CTTGTGTGCTGGATTCTCCTTTGGTCTGATTAGGCTG |
| RIG-I K270A Fw | RIG-I K270A | CTACAGGTTGTGGAGCAACCTTTGTTTCAC |
| RIG-I K270A Rev | RIG-I K270A | GTGAAACAAAGGTTGCTCCACAACCTGTAG |
| RIG-I K861A/K858A/K851A | RIG-I K861A/K858A/K851A | AAGTTTTGAAGCAAGAGCAGCGATATTCTGTGCCCGACAGAACTGC |
| RIG-I K861A/K858A/K851A | RIG-I K861A/K858A/K851A | GAAAACTGCGCTGGCTTGGGATGTGGTCTACTCACAAAGCATTCC |

**Supplementary Table 5. Sequences of internally truncated vRNA templates used in RNP assays.**

| TEMPLATE NAME | VRNA-SENSE SEQUENCE (5′- 3′) |
|---|---|
| NA76 | AGUAGAAACAAGGAGUUUUUUGAACAAACUACUUGUCAAUUUCUGGUUUGGAUUCAUUUAAACUCCUGCUUUUGCU |
| NA124 | AGUAGAAACAAGGAGUUUUUUGAACAAACUACUUGUCAAUGGUGAACGGGAGCUCAGCACCGUACAGAUCGAUCCAAUGGUUAUUAUUUU<br>CUGGUUUGGAUUCAUUUAAACUCCUGCUUUUGCU |
| NA196 | AGUAGAAACAAGGAGUUUUUUGAACAAACUACUUGUCAAUGGUGAACGGGAGCUCAGCACCGUCUGGCCAAGACCAAUCUACAGUAUCACC<br>AUUCACACUAUUUGCAAUAUUAGGCUAAUUAUUCCGACUACCAUACAGAUCGAUCCAAUGGUUAUUAUUUUCUGGUUUGGAUUCAUUUAA<br>ACUCCUGCUUUUGCU |
| NA244 | AGUAGAAACAAGGAGUUUUUUGAACAAACUACUUGUCAAUGGUGAACGGGAGCUCAGCACCGUCUGGCCAAGACCAAUCUACAGUAUCACC<br>AUUCACACCACAAAAAGAAAUGAUGCUCCCACAAUCCAUAUUGAGAUUAUAUUUCCUAUUUGCAAUAUUAGGCUAAUUAUUCCGACUACCA<br>UACAGAUCGAUCCAAUGGUUAUUAUUUUCUGGUUUGGAUUCAUUUAAACUCCUGCUUUUGCU |
| NP76 | AGUAGAAACAAGGGUAUUUUUCUUUACUAGUGACUUCGAUGUCACUCUGUGAGUACUAGUCUACCCUGCUUUUGCU |
| NP125 | AGUAGAAACAAGGGUAUUUUUCUUUAAUUGUCGUACUCCUCUGCAUUGUCACUAGUUCGUUUGGUGCCUUUGGUCGCCAUGAUUUCGAU<br>GUCACUCUGUGAGUACUAGUCUACCCUGCUUUUGCU |
| NP197 | AGUAGAAACAAGGGUAUUUUUCUUUAAUUGUCGUACUCCUCUGCAUUGUCUCCGAAGAAAUAAGAUCCUUCAUUACUCAUGUCAAAGGAG<br>GGCACGAUCGGGCUCGUUGCACUAGUCUGUUCGUAAGAUCGUUUGGUGCCUUUGGUCGCCAUGAUUUCGAUGUCACUCUGUGAGUACUA<br>GUCUACCCUGCUUUUGCU |
| NP246 | AGUAGAAACAAGGGUAUUUUUCUUUAAUUGUCGUACUCCUCUGCAUUGUCUCCGAAGAAAUAAGAUCCUUCAUUACUCAUGUCAAAGGAG<br>GGCACGAUCGGGCUCGUUGCCUUUUCGUCCGAGAGCUCGAAGACUCCCCGCCCCUGGAAAGACACUAGUCUCCAUCUGUUCGUAAGAUCGU<br>UUGGUGCCUUUGGUCGCCAUGAUUUCGAUGUCACUCUGUGAGUACUAGUCUACCCUGCUUUUGCU |

**Supplementary Table 6. Primers used for primer extension.**

| PRIMER NAME | TARGET RNA | PRIMER SEQUENCE (5′- 3′) |
|---|---|---|
| 5S 100 | 5S rRNA | TCCCAGGCGGTCTCCCATCC |
| NP 149- | NP vRNA | ATTTCTTCGGAGACAATGCAG |
| NP 149+ | NP c/mRNA internal | TAAGATCGTTTGGTGCCTTTG |
| NA 1280 | NA vRNA internal | TGGACTAGTGGGAGCAT |
| NA 160 | NA c/mRNA internal | TCCAGTATGGTTTTGATTTCC |
| PB1 vRNA | PB1 vRNA internal | TGATTTCGAATCTGGAAGGA |
| PB1 c/mRNA | PB1 c/mRNA internal | TCCATGGTGTATCCTGTTCC |
| HA vRNA | HA vRNA internal | TACTCAACTGTCGCCAGTTC |
| HA c/mRNA | HA c/mRNA internal | GTCACTGCCACATTCTTCTC |
| M vRNA | M vRNA internal | GAAAAGAGGGCCTTCTACGG |
| M c/mRNA | M c/mRNA internal | AGCCATTCCATGAGAACCTC |
| NA 5′ | NA cRNA terminal | AGTAGAAACAAGGAGTTTTTTG |
| NA-2 | NA vRNA terminal | AGCGAAAGCAGGAGTTTAAATG |
| NA c/mRNA mini | NA c/mRNA internal (for short templates) | CTACTTGTCAATGGTGAACG |
| NP 5′ | NP cRNA terminal | AGTAGAAACAAGGGTATTTTTC |
| GC- | NP vRNA terminal | AGCAAAAGCAGGGTAGACTAGT |
| NP c/mRNA mini | NP c/mRNA internal (for short templates) | GTCGTACTCCTCTGCATTG |
| $VNdT_{20}$ | Polyadenylated RNA | VNTTTTTTTTTTTTTTTTTTTT |

V for Adenine, Guanine or Cytosine

N for Adenine, Guanine, Cytosine or Thymine

**Supplementary Table 7. RNA templates used for polymerase activity assays.**

| NAME | SEQUENCE (5′- 3′) |
|---|---|
| AG11_primer for capping | ppGAAUACUCAAG |
| vRNA_5p | AGUAGAAACAAGGCC |
| vRNA_3p | GGCCUGCUUUUGCU |

| | |
|---|---|
| NA71 | AGUAGAAACAAGGAGUUUUUUGAACAAACUACUUGUCAAUGGUUGGAUUCAUUUAAACUCCUGCUUUUGCU |
| NA71-U | AGUAGAAACAAGGAGAUGUGUGAACAAACUACUUGUCAAUGGUUGGAUUCAUUUAAACUCCUGCUUUUGCU |
| NP71 | AGUAGAAACAAGGGUAUUUUUCUUUACUAGUUAGGUAGUAUACCUAGUAACUAGUCUACCCUGCUUUUGCU |
| NP71-U | AGUAGAAACAAGGGAAUGUGUCUUUACUAGUUAGGUAGUAUACCUAGUAACUAGUCUACCCUGCUUUUGCU |

**Supplementary Table 8. Primers used for mvRNA and 5S rRNA RT-PCR.**

| PRIMER | TARGET | STEP | PRIMER SEQUENCE (5′- 3′) |
|---|---|---|---|
| Lv3aa | mvRNA | RT | GTTCAGACGTGTGCTCTTCCGATCTAGC+A+AAAGCAGG |
| Lv3ga | mvRNA | RT | GTTCAGACGTGTGCTCTTCCGATCTAGCG+AAAGCAGG |
| Lv5 | mvRNA | 2nd strand | CACGACGCTCTTCCGATCTHNNNNNNNAGTAGAA+A+CAAGG |
| P7 | mvRNA | PCR | GACGTGTGCTCTTCCGATCT |
| P5_IRdye800 | mvRNA | PCR | CACGACGCTCTTCCGATCT |
| 5S 100 | 5S rRNA | RT | TCCCAGGCGGTCTCCCATCC |
| 5S_Fw | 5S rRNA | 2nd strand and PCR | GTCTACGGCCATACCACC |
| 5S100_Rev_ATTO647N | 5S rRNA | PCR | TCCCAGGCGGTCTCCCATCC |

+ for LNA bases

H for Adenine, Cytosine or Thymine

N for Adenine, Guanine, Cytosine or Thymine

**Supplementary Table 9. Primers/oligos used for TSO-based RT-PCR and qPCR.**

| PRIMER/OLIGO | TARGET | STEP | PRIMER SEQUENCE (5′- 3′) |
|---|---|---|---|
| Tuni-13 | cRNA, ccRNA | RT | ACGCGTGATCAGTAGAAACAAGG |
| Tuni-13 LNA3 | cRNA, ccRNA | RT | ACGCGTGATCAGTAGAAA+CA+AG+G |
| Oligo $d(T)_{20}$ | mRNA | RT | TTTTTTTTTTTTTTTTTTTT |
| TSO | N/A | RT | GCTAATCATTGCAAGCAGTGGTATCAACGCAGAGTACATrGrGrG |
| TSO Fw | TSO | PCR | CATTGCAAGCAGTGGTATCAAC |
| PB2 Rev | PB2 | PCR | CTGCGACATTAGATTCCTTAGTTC |
| PB1 Rev | PB1 | PCR | GTAAAGTCGGATTGACATCCATTC |
| PA Rev | PA | PCR | GGATTGAAGCATTGTCGCAC |
| HA Rev | HA | PCR | CAGGACTAGTACAAAAGCCTTC |
| NP Rev | NP | PCR | GATTTCGATGTCACTCTGTGAG |
| NA Rev | NA | PCR | GATCCAATGGTTATTATTTTCTGGTTTG |
| M Rev | M | PCR | GACCTCGGTTAGAAGACTCATC |
| NS Rev | NS | PCR | GCTTGACACAGTGTTTGGATC |
| 18S rRNA Fw | 18S rRNA | qPCR | ACCCGTTGAACCCCATTGGTGA |
| 18s rRNA Rev | 18S rRNA | RT/qPCR | GCCTCACTAAACCATCCAATCGG |
| NA qPCR Fw | NA | qPCR | GTTTGAATCGGTTGCTTGG |
| NA qPCR Rev | NA | qPCR | CTGCTCCATCATCTGGACC |

+ for LNA bases

r for ribonucleotides

**Supplementary Table 10. Primers/oligos used for TSO-based RT-PCR and NGS library preparation.**

| PRIMER/OLIGO | TARGET | STEP | PRIMER SEQUENCE (5′- 3′) |
|---|---|---|---|
| 3′ cRNA primer | cRNA, ccRNA | RT | ATATGGTCTCGTATTAGTAGAAACAAGG |
| TSO 2 | N/A | RT | Biotin-AAGCAGTGGTATCAACGCAGAGTACATrNrG+G |
| TSO-specific circularization primer | TSO | PCR of 5’ region | AATACGAGACCATATAAGCAGTGGTATCAACGCAGAGT |
| Ba-PB2-2341R | PB2 | PCR of 5’ region | ATATGGTCTCGTATTAGTAGAAACAAGGTCGTTT |

| | | | |
|---|---|---|---|
| Bm-PB1-2341R | PB1 | PCR of 5’ region | ATATGGTCTCGTATTAGTAGAAACAAGGCATTT |
| Bm-PA-2233R | PA | PCR of 5’ region | ATATGGTCTCGTATTAGTAGAAACAAGGTACTT |
| Bm-NS-890R | HA/NS | PCR of 5’ region | ATATGGTCTCGTATTAGTAGAAACAAGGGTGTTTT |
| Bm-NP-1565R | NP | PCR of 5’ region | ATATGGTCTCGTATTAGTAGAAACAAGGGTATTTTT |
| Ba-NA-1413R | NA | PCR of 5’ region | ATATGGTCTCGTATT AGTAGAAACAAGGAGTTTTTT |
| Bm-M-1027R | M | PCR of 5’ region | ATATGGTCTCGTATTAGTAGAAACAAGGTAGTTTTT |
| NSrnd1UP | NS | NS PCR | TGCCTCATCAGATTCTTCCTTC |
| NSrnd1DWN | NS | NS PCR | GCAGTAATGAGAATGGGAGACC |
| NSrnd2UP | NS | Append partial Illumina adapter | TCGTCGGCAGCGTCAGATGTGTATAAGAGACAGAGTG CTGCCTCTTCCTCTTA |
| NSrnd2DWN | NS | Append partial Illumina adapter | GTCTCGTGGGCTCGGAGATGTGTATAAGAGACAGGGA ACAATTAGGTCAGAAGTTTGAGG |

+ for LNA bases

r for ribonucleotides

N for Adenine, Guanine, Cytosine or Thymine

**Supplementary Table 11. Primers/oligos used for *in vitro* transcription.**

| PRIMER/OLIGO | PRODUCT | PRIMER SEQUENCE (5′- 3′) |
|---|---|---|
| NA T7 ccRNA/mRNA Fw | ccRNA/mRNA | TAATACGACTCACTATAGGGAATACTCAAGGCAAAAGCAGGAGTT TAAATG |
| NA T7 vRNA/svRNA Fw | vRNA/svRNA | TAATACGACTCACTATTAGTAGAAACAAGGAGTTTTTTGAAC |
| NA T7 cRNA Fw | cRNA | TAATACGACTCACTATTAGCAAAAGCAGGAGTTTAAATG |
| NA cRNA/ccRNA rev (NA 5′) | cRNA/ccRNA | AGTAGAAACAAGGAGTTTTTTG |
| NA mRNA Rev | mRNA | TTTTTTTTTTTTTTTTTTTTTTTTTTTTTTGAACAAACTACTTGTCAAT GGTG |
| NA ccRNA-3′ Rev | ccRNA-3′ | AAACTACTTGTCAATGGTGAACG |
| NA vRNA Rev | vRNA | AGCAAAAGCAGGAGTTTAAATG |
| NA svRNA rev | svRNA | GTTCAAAAAACTCCTTGTTTCTACTAATAGTGAGTCGTATTA |

**Supplementary Table 12. Sequences of *in vitro* transcription products.**

| PRODUCT NAME | SEQUENCE (5′- 3′) |
|---|---|
| NA196 vRNA | AGUAGAAACAAGGAGUUUUUUGAACAAACUACUUGUCAAUGGUGAACGGGAGCUCAGCACCGUCUGGCCAAGACCAAUCUACAGUAUCACCAUUCACACUAUUUGCAAUAUUAGGCUAAUUAUUCCGACUACCAUACAGAUCGAUCCAAUGGUUAUUAUUUUCUGGUUUGGAUUCAUUUAAACUCCUGCUUUUGCU |
| NA196 cRNA | AGCAAAAGCAGGAGUUUAAAUGAAUCCAAACCAGAAAAUAAUAACCAUUGGAUCGAUCUGUAUGGUAGUCGGAAUAAUUAGCCUAAUAUUGCAAAUAGUGUGAAUGGUGAUACUGUAGAUUGGUCUUGGCCAGACGGUGCUGAGCUCCCGUUCACCAUUGACAAGUAGUUUGUUCAAAAAACUCCUUGUUUCUACU |
| NA196 mRNA | GGGAAUACUCAAGGCAAAAGCAGGAGUUUAAAUGAAUCCAAACCAGAAAAUAAUAACCAUUGGAUCGAUCUGUAUGGUAGUCGGAAUAAUUAGCCUAAUAUUGCAAAUAGUGUGAAUGGUGAUACUGUAGAUUGGUCUUGGCCAGACGGUGCUGAGCUCCCGUUCACCAUUGACAAGUAGUUUGUUCAAAAAAAAAAAAAAAAAAAAAAAAAAAAAAA |
| NA196 ccRNA | GGGAAUACUCAAGGCAAAAGCAGGAGUUUAAAUGAAUCCAAACCAGAAAAUAAUAACCAUUGGAUCGAUCUGUAUGGUAGUCGGAAUAAUUAGCCUAAUAUUGCAAAUAGUGUGAAUGGUGAUACUGUAGAUUGGUCUUGGCCAGACGGUGCUGAGCUCCCGUUCACCAUUGACAAGUAGUUUGUUCAAAAAACUCCUUGUUUCUACU |
| NA196 ccRNA-3′ | GGGAAUACUCAAGGCAAAAGCAGGAGUUUAAAUGAAUCCAAACCAGAAAAUAAUAACCAUUGGAUCGAUCUGUAUGGUAGUCGGAAUAAUUAGCCUAAUAUUGCAAAUAGUGUGAAUGGUGAUACUGUAGAUUGGUCUUGGCCAGACGGUGCUGAGCUCCCGUUCACCAUUGACAAGUAGUUU |
| NA svRNA | AGUAGAAACAAGGAGUUUUUUGAAC |
