# Supplementary material for: Influenza A virus transcription generates capped cRNAs that activate RIG-I
Source: bioRxiv. 2024 Nov 12:2024.11.12.623191. Preprint. [Version 1] doi: 10.1101/2024.11.12.623191 (PMC11601390; doi:10.1101/2024.11.12.623191)
Supplement: Supplementary file 3 [file media-3.pdf]

Supplementary Figures

Influenza A virus transcription generates capped cRNAs that activate RIG-I

Elizaveta Elshina[1,2], Emmanuelle Pitre[1,2], Marisa Mendes[3], Brandon Schweibenz[4], Hollie French[2,#], Ji Woo Park[1], Wei Wang[5], Joseph Marcotrigiano[4], Alistair B. Russell[3], Aartjan J.W. te Velthuis[1,*]

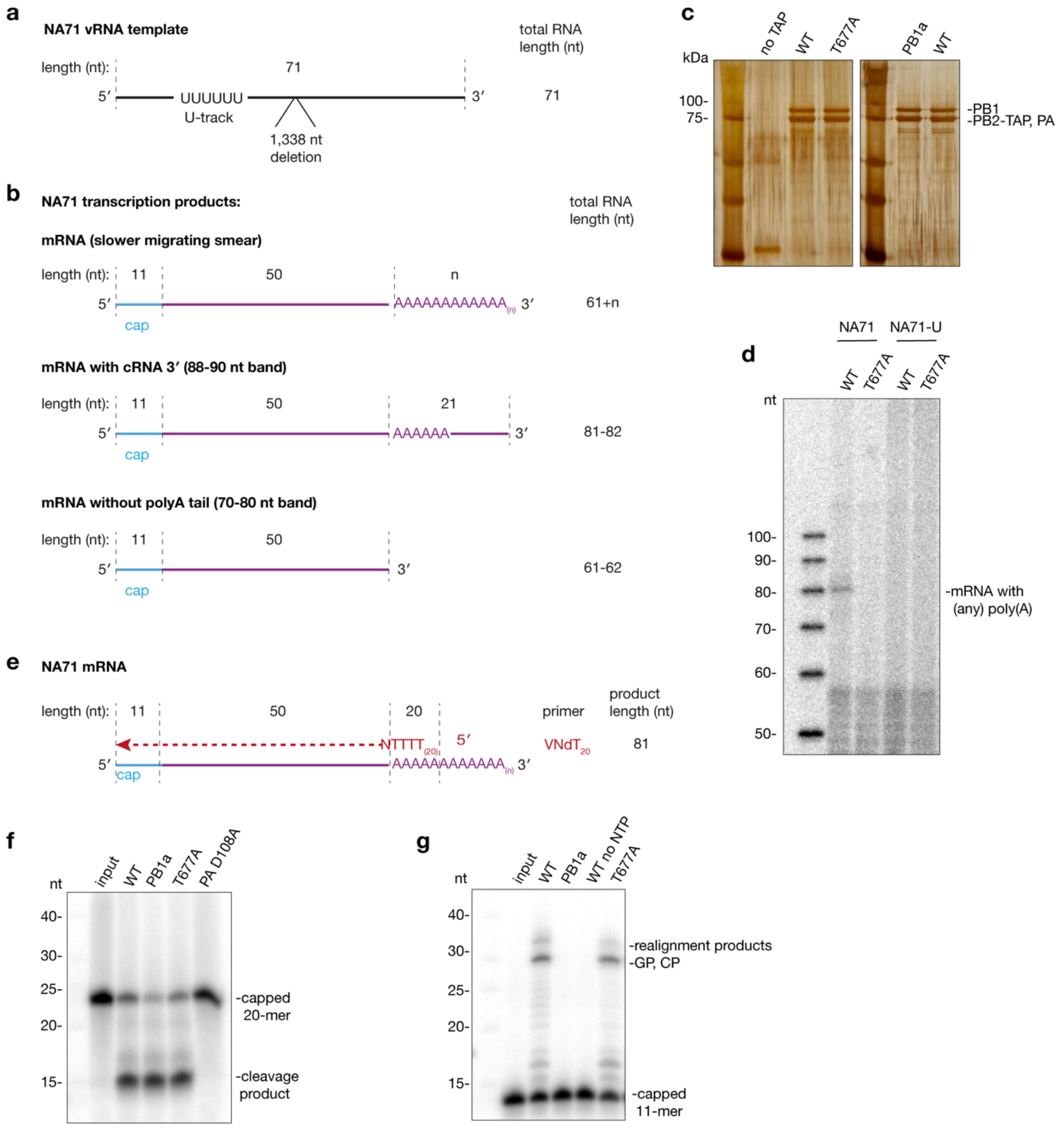

Supplementary Fig. 1| *In vitro* activity assays with purified polymerases. a, Schematic of NA71 vRNA template used for transcription assay in Fig. 1c and Fig. 1d. **b**, Schematic of proposed transcription products observed in Fig. 1c. **c**, Purified IAV polymerases visualised on a silver-stained SDS-PAGE gel. The subunits of the IAV RNA polymerase are indicated. The cleaved PB2-TAP subunit runs close to the PA subunit after TEV cleavage of the TAP tag. **d**, Primer extension assay using $VNdT_{20}$ primer and RNA isolated from transcription assay in the presence of the templates NA71 and NA71-U. **e**, Schematic of primer extension with $VNdT_{20}$ primer. **f**, *In vitro* cap cleavage assay of a capped 20-mer in the presence of the model vRNA promoter (vRNA_5p and vRNA_3p; Supplementary Table 7). The PA D108A endonuclease mutant was used as a negative control. **g**, *In vitro* transcription initiation assay with 11-mer capped primer in the presence of the model vRNA promoter. The 'no NTP' condition and an inactive polymerase mutant (PB1a) were used as negative controls. Transcription initiation products from positions 2C (CP) and 3G (GP) as well as realignment products are indicated.

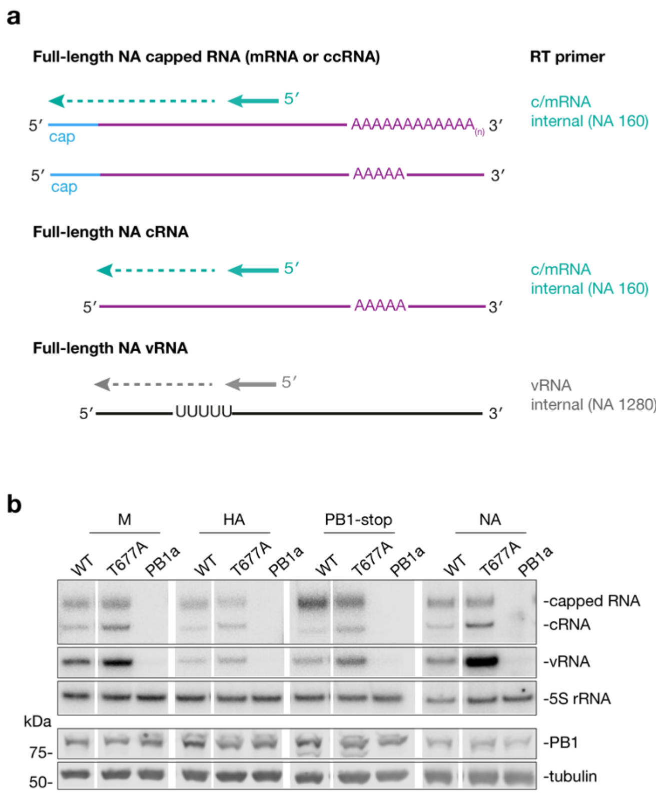

Supplementary Fig. 2| RNP reconstitution assay with full-length templates. **a**, Schematic of the primer extension assay for the full-length segment 6 (NA) using internal c/mRNA and vRNA primers. **b**, Representative primer extension gel images are shown for full-length M, HA, PB1 with a start codon mutation (PB1-stop), and NA templates (top). Primers used were: M vRNA and M c/mRNA; HA vRNA and HA c/mRNA; PB1 vRNA and PB1 c/mRNA; NA 1280 and NA 160. Western blot analysis of PB1 expression is shown (bottom two rows).

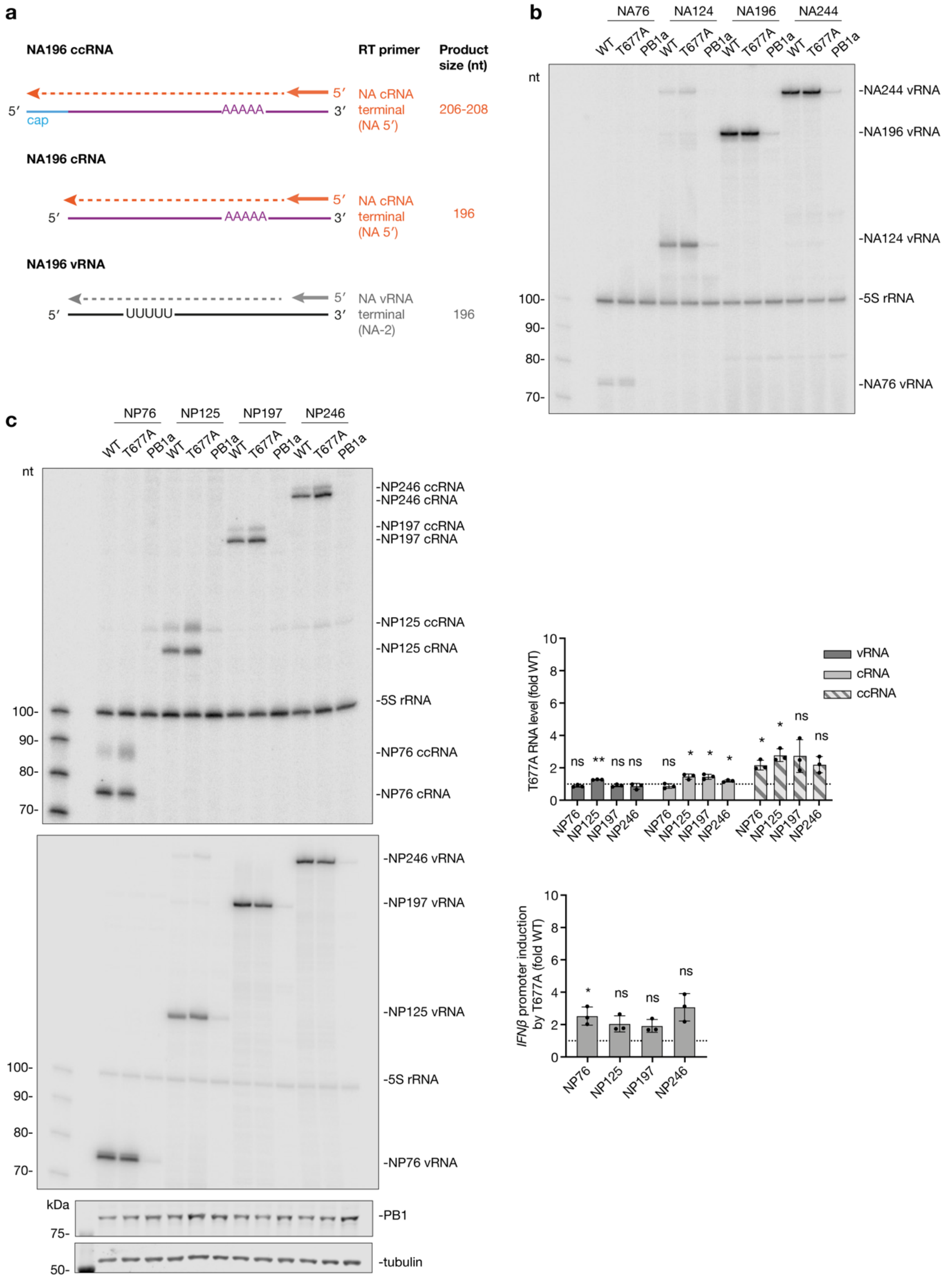

Supplementary Fig. 3| RNP reconstitution assays with short templates. a, Schematic of primer extension assay for short segment 6-based templates using the NA196 template as an example. Primer binding sites and sizes of expected products are indicated. **b**, RNP reconstitution assay with short segment 6-based templates. The panel shows a representative primer extension gel image with a terminal vRNA primer (NA-2). The quantification of three independent replicates is shown in Fig. 1f. **c**, RNP reconstitution assay with short segment 5-based templates and subsequent primer extension analysis utilising the terminal cRNA (NP 5′) and vRNA (GC-) primers.

Representative primer extension gel images (top two images) and western blot analysis of protein expression (bottom two images) are shown. The quantification of the vRNA, cRNA and ccRNA levels produced by the T677A relative to the wild-type polymerase are shown in the top right graph. The quantification of *IFN-β* promoter activity is shown in the bottom right graph. Data in the graphs of panel **c** are shown as mean ± SD from three independent experiments. Statistical significance was determined using a one-sample t-test; (ns=non-significant, **P*<0.05, ***P*<0.01).

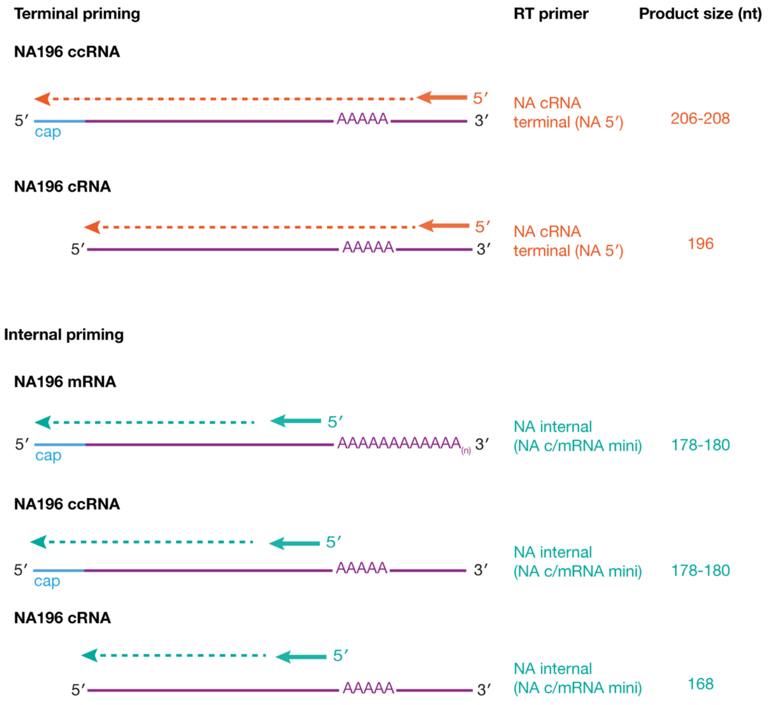

Supplementary Fig. 4| Schematic of the primer extension assay with the terminal and internal primers used in Fig. 1g and h. Primer binding sites and the sizes of expected products are indicated.

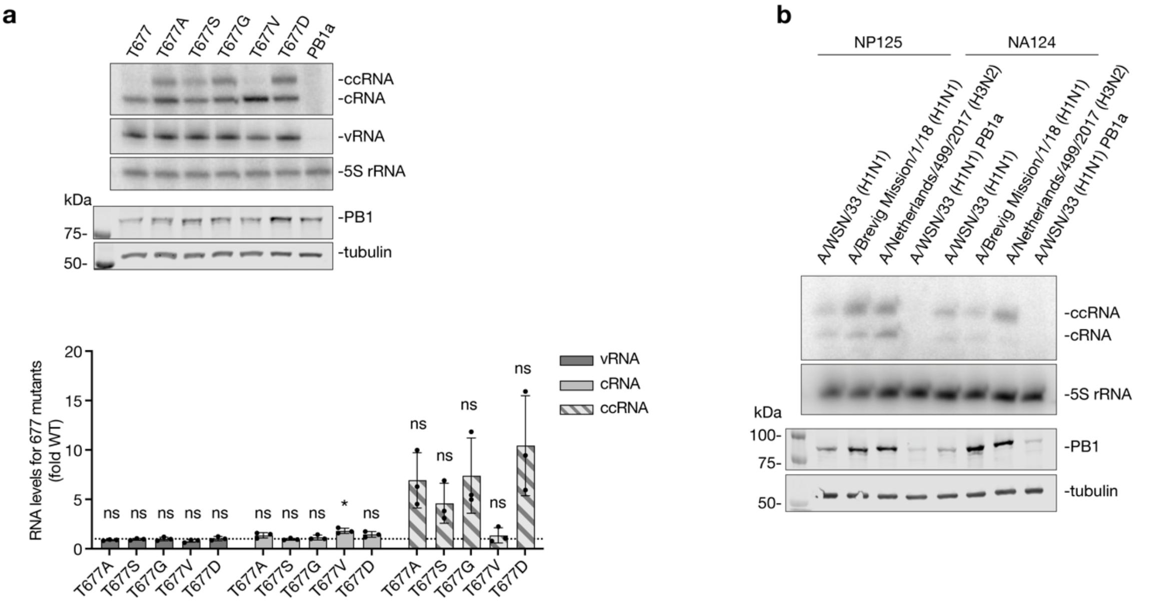

Supplementary Fig. 5| ccRNA production by A/WSN/33 polymerase with different PB1 T677 mutations and ccRNA production by the IAV polymerases of different IAV strains. a, RNP assay using the NA196 template and A/WSN/33 polymerases with different PB1 T677 amino acid substitutions. Primer extension analysis with terminal cRNA (NA 5′) and vRNA (NA-2) primers (top three images) and western blot analysis of PB1 expression levels (top images four and five) are shown. The quantification of vRNA, cRNA, and ccRNA levels produced by T677A mutant relative to the wild-type polymerase is shown in the bottom graph. Data are shown as mean ± SD from three independent experiments. Statistical significance was determined using a one-sample t-test; (ns=non-significant, *P<0.05). **b**, RNP assay analysis with the segment 5-derived NP125 and segment 6-derived NA124 templates and the RNA polymerases of different IAV strains. The primer extension analysis was performed with terminal cRNA primers (NP 5′ or NA 5′). The primer extension analysis shown is representative of two experiments.

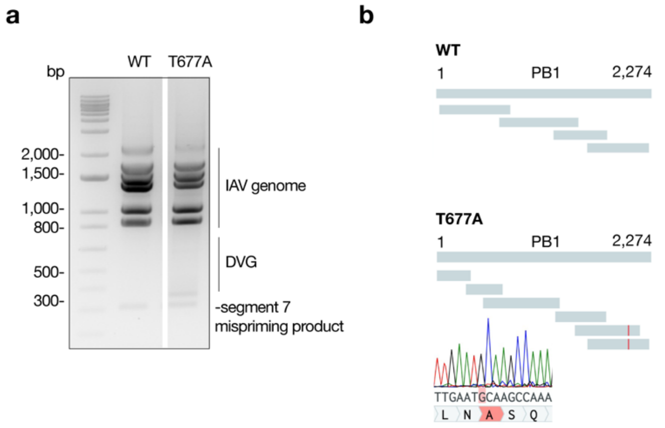

Supplementary Fig. 6| Generation of wild-type and T677A A/WSN/33 virus stocks. a, IAV stocks were generated by reverse genetics and plaque purified. To assess the DVG content in the virus preparations, RT-PCR was performed on viruses isolated from 12 individual plaques using universal Tuni-12 and Tuni-13 primers, which bind to 3' vRNA and cRNA termini, respectively. Virus isolates with the lowest DVG content were selected and used for subsequent infection experiments. The 300 bp band observed was isolated, sequenced and identified as a mispriming product of the Tuni-12 primer to an internal sequence of segment 7. **b**, Validation of the PB1 sequence in the viral stocks was performed by Sanger sequencing. The schematic shows the areas covered by different sequence runs. The sequence trace of the T677A mutant virus is shown at the bottom of the image.

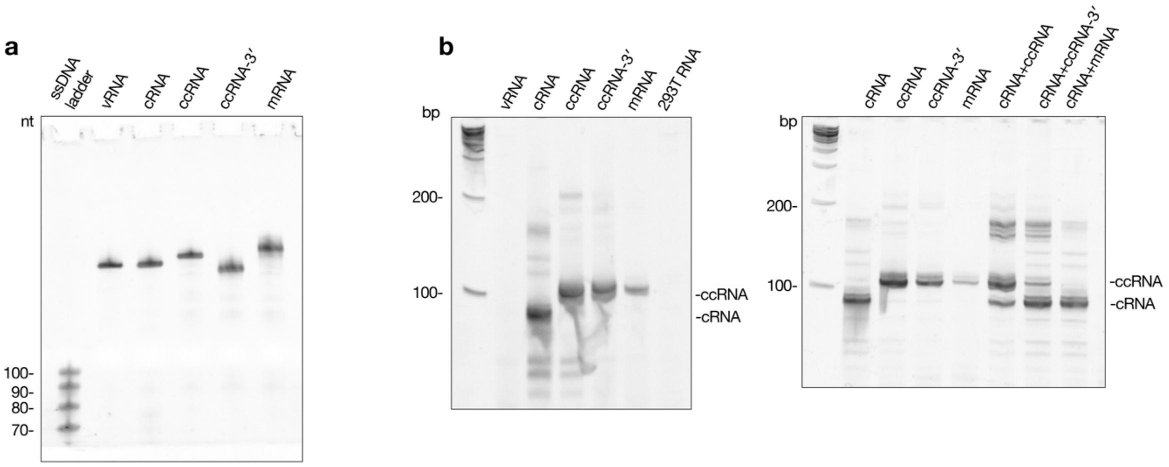

Supplementary Fig. 7| Optimisation of the TSO-based RT-PCR using *in vitro* transcribed (IVT) RNA. a, *In vitro* transcription was performed on NA196-based PCR products to generate vRNA, cRNA, ccRNA, ccRNA lacking 25 3'-terminal nt (ccRNA-3'), and mRNA molecules. Transcripts were gel purified. Next, the ccRNA, ccRNA-3' and mRNA molecules were capped and methylated, and any remaining uncapped RNA enzymatically digested. The RNA quality was evaluated using denaturing PAGE with 100 ng of RNA per lane. A representative image of the analysis of the prepared RNAs is shown. **b**, Gel electrophoresis of the TSO-based RT-PCR products using the IVT RNA samples as input RNA. Total RNA from HEK293T cells was included as a negative control. The expected cRNA and ccRNA products are indicated. The other bands derive from RT or PCR amplification errors.

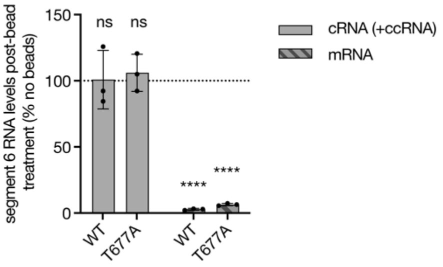

Supplementary Fig. 8| Assessment of efficiency of mRNA depletion by oligo d(T)$_{25}$-bead treatment. Total RNA from A549 infected with wild-type or T677A viruses was subjected to oligo d(T)$_{25}$-bead treatment or left untreated. The segment 6 cRNA and mRNA levels were quantified by qRT-PCR, using a terminal cRNA primer (which also detects ccRNA) and oligo d(T)$_{20}$ primer, respectively. Data are shown as mean ± SD from three independent experiments. Statistical significance was determined using a one-sample t-test; (ns=non-significant, ****P<0.0001).

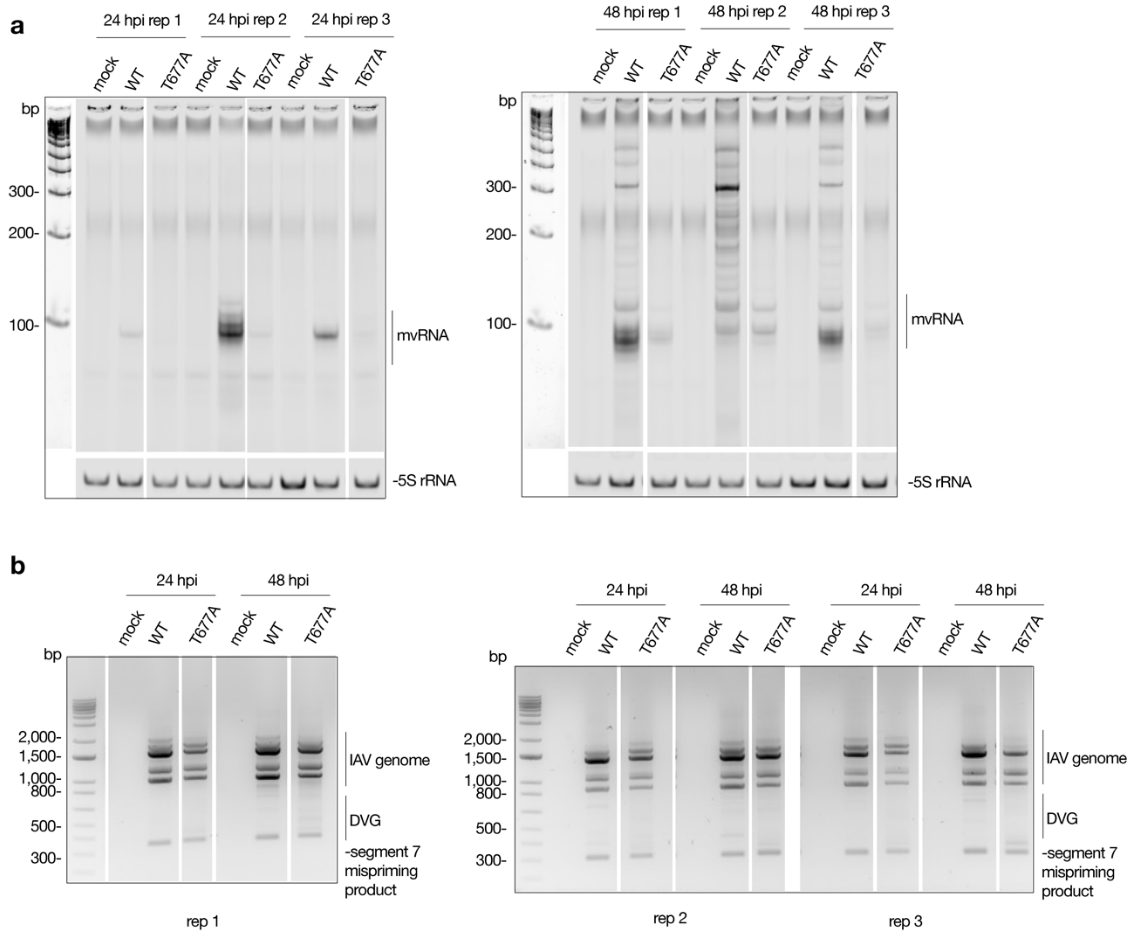

Supplementary Fig. 9|Generation of non-canonical replication products by wild-type and T677A viruses. a, RT-PCR analysis of mvRNA synthesis was performed using RNA extracted from HEK293-luc cells infected with the wild-type or T677A viruses at an MOI of 0.01 for 24 or 48 hours. Total RNA from three independent replicates was size-fractionated, and the small fraction (<200 nt) was used for RT-PCR with universal primers targeting the conserved genome segment termini. 5S rRNA was used as a cellular RNA loading control. **b**, DVG production was assessed in the same infection samples as in panel **a**, using total RNA and RT-PCR with universal primers.

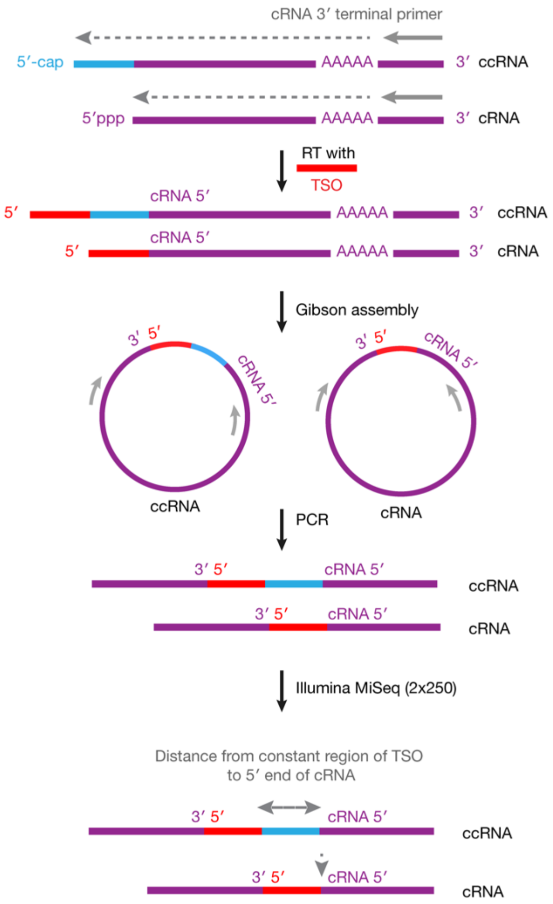

Supplementary Fig. 10| Schematic of TSO-based RT-PCR combined with NGS for analysis of 5' cRNA termini. RT was done with a universal cRNA 3’ primer, which binds to the conserved 3' cRNA termini of all eight segments, in the presence of TSO to allow the RT to template-switch at the 5' cRNA terminus. The TSO sequence was thus appended by the RT enzyme to the 5' termini of the cDNA molecules produced. The RT step was followed by Gibson assembly, which ligated the 3' and 5' cDNA termini. Next, PCR was done using segment-specific internal primers and the resulting products were sequenced using 2x250 Illumina MiSeq. The ccRNA and cRNA molecules were identified by measuring the distance between the constant sequence of the TSO and the 5' end of cRNA. Molecules with 8-14 nt between the TSO and the 5' end of cRNA were considered ccRNAs.

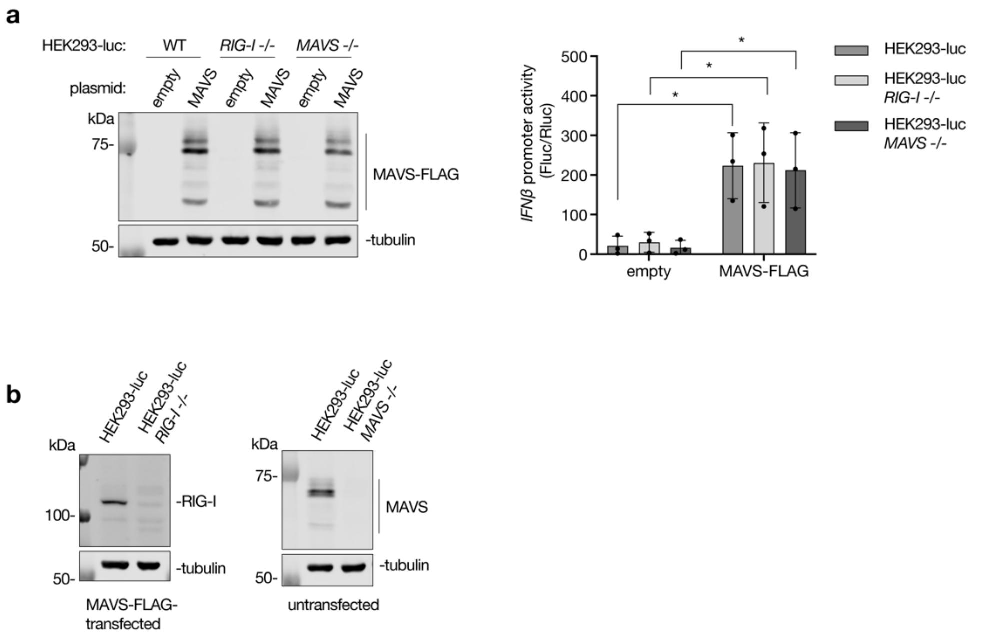

Supplementary Fig. 11|Characterisation of the *RIG-I* -/- and *MAVS* -/- HEK293-luc cell lines. a, Wild-type, *RIG-I* -/-, and *MAVS* -/- HEK293-luc cells were transfected with either a MAVS-FLAG-expressing plasmid or an empty control plasmid, along with a *Renilla* luciferase reporter plasmid, for 24 hours. MAVS-FLAG expression was assessed by western blotting (left). The IFN-β luciferase reporter activity was expressed as a proportion of *Renilla* luciferase signal (right). Data are presented as mean ± SD from three independent experiments. Statistical significance was determined using two-way ANOVA with Šídák's multiple comparisons test (**P*<0.05). **b**, Efficiency of *RIG-I* and *MAVS* knockout was evaluated by western blotting. RIG-I expression in the left panel was induced by MAVS-FLAG overexpression for 24 hours.

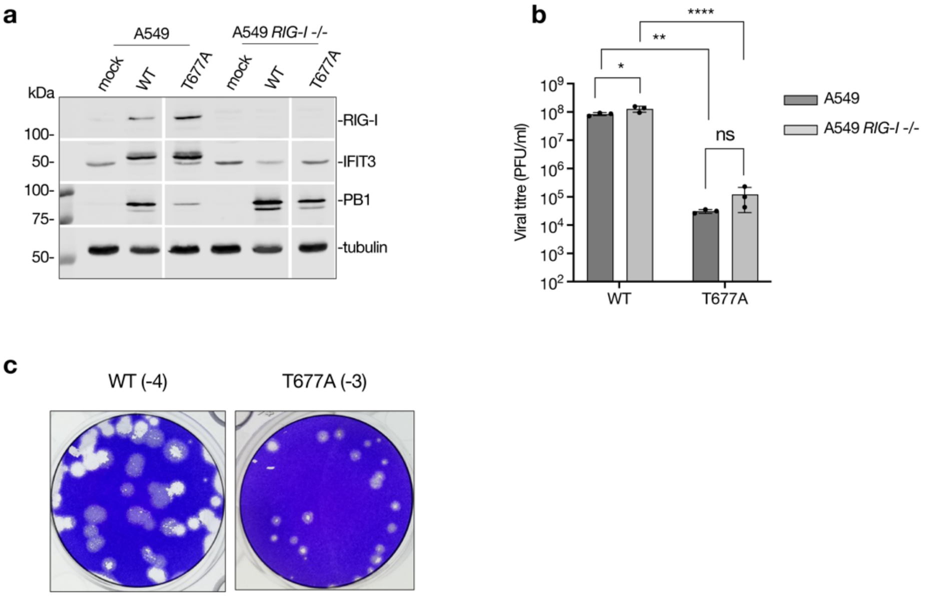

Supplementary Fig. 12| Characterisation of T677A virus growth and innate immune activation. a, A549 or A549 *RIG-I* -/- cells were infected with wild-type or T677A viruses at an MOI of 0.01, or mock infected, for 48 hours. ISG and PB1 expression levels were assessed by western blotting. A representative image from three independent experiments is shown. **b**, Viral titres in cell culture supernatants, collected as described in panel **a**, were measured by plaque assay. Data are presented as mean ± SD from three independent experiments. Statistical significance was determined using two-way ANOVA with Tukey's multiple comparisons test (ns=non-significant, *P<0.05, **P<0.01, ****P<0.0001). **c**, Representative images from three independent experiments showing plaque sizes of viruses collected from infected HEK293-luc cells and grown on an MDCK monolayer. Sample dilutions are indicated.

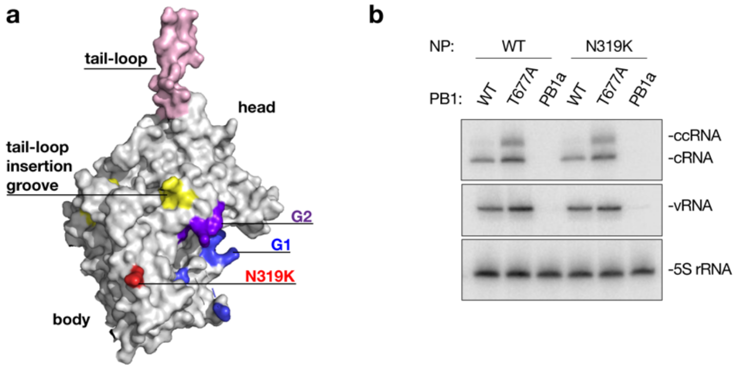

Supplementary Fig. 13| Characterisation of N319K mutation in NP. a, Location of the N319K mutation in the structure of IAV NP (PDB: 2Q06). The structural features of NP, including RNA-binding G1 and G2 regions, tail-loop, and tail-loop insertion groove are indicated. **b**, RNP reconstitution assay was performed using wild-type or N319K NP and wild-type or T677A PB1, with the NA196 template. Primer extension analysis was done using primers targeting the 3' termini of cRNA/ccRNA (NA 5′) and vRNA (NA-2) molecules.

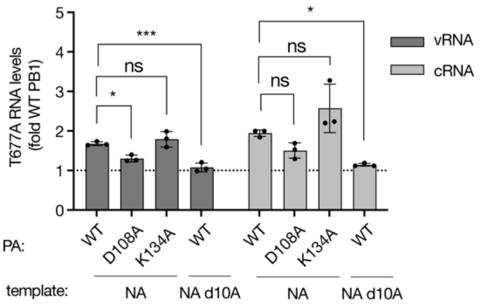

Supplementary Fig. 14| The role of transcription in IFN activation by T677A mutant polymerase. Quantification of cRNA and vRNA levels produced by T677A relative to the wild-type polymerase in the RNP assay described in Fig. 3g. Data are presented as mean ± SD from three independent experiments. Statistical significance was determined using one-way ANOVA with Dunnett's multiple comparisons test (ns=non-significant, *P<0.05, ***P<0.001).

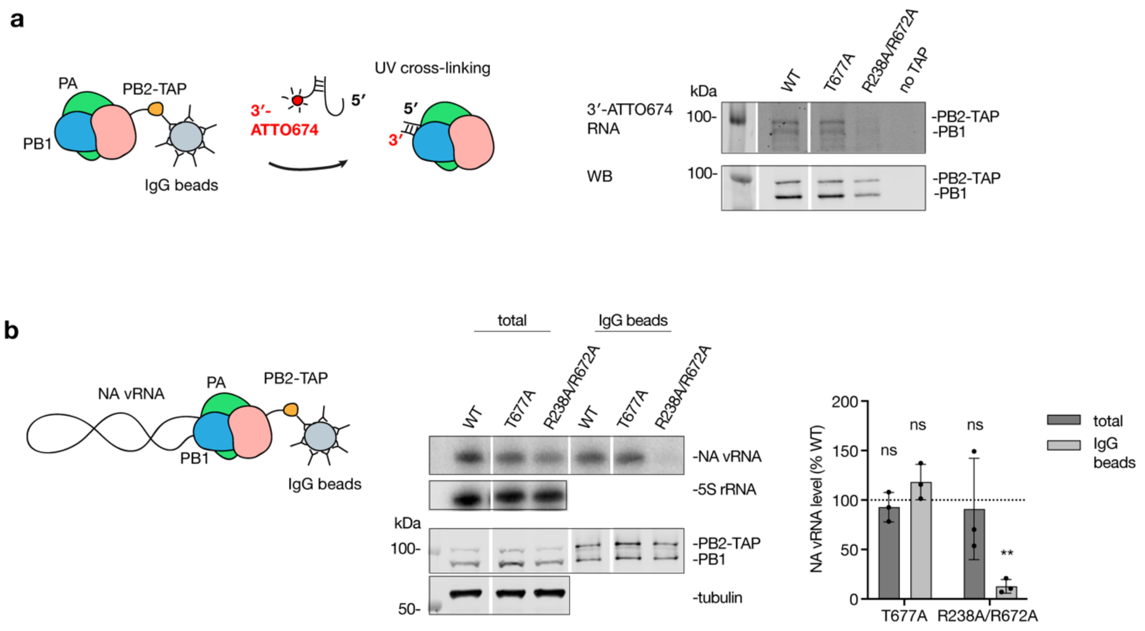

Supplementary Fig. 15| Assessment of binding between the wild-type or T677A mutant polymerases and the viral promoter. a, Schematic of the promoter-binding assay (left). Wild-type or T677A mutant polymerases with a PB2-TAP tag were expressed in 293T cells and cell lysates were incubated with IgG beads. The PB1 R238A/R672A RNA-binding mutant and a PB2 subunit lacking the TAP tag were used as negative controls. The polymerases, immobilized on beads, were then incubated with pre-annealed 3' and 5' ends of the vRNA promoter with the 1st nucleotide of the 3' end labelled with ATTO-674N. Covalent linkage of the viral promoter to the polymerase was achieved using 254 nm UV light. The samples were analyzed by SDS-PAGE (right) and fluorescently labelled RNA visualized at 700 nm. The same gel was probed by western blotting with anti-PB1 antibodies, which also detect the uncleaved TAP-tag on PB2. **b**, Schematic of the full-length vRNA binding assay. PB1 wild-type, T677A, or R238A/R672A mutant polymerases containing a PB2-TAP tag were co-expressed with full-length segment 6 (NA) vRNA template in HEK293T cells. Polymerases were captured on IgG beads and levels of co-precipitating NA vRNA were assessed by primer extension analysis with NA 1280 primer (right). Data are presented as mean ± SD from three independent experiments. Statistical significance was determined using one-sample t-test (ns=non-significant, ***P*<0.01).

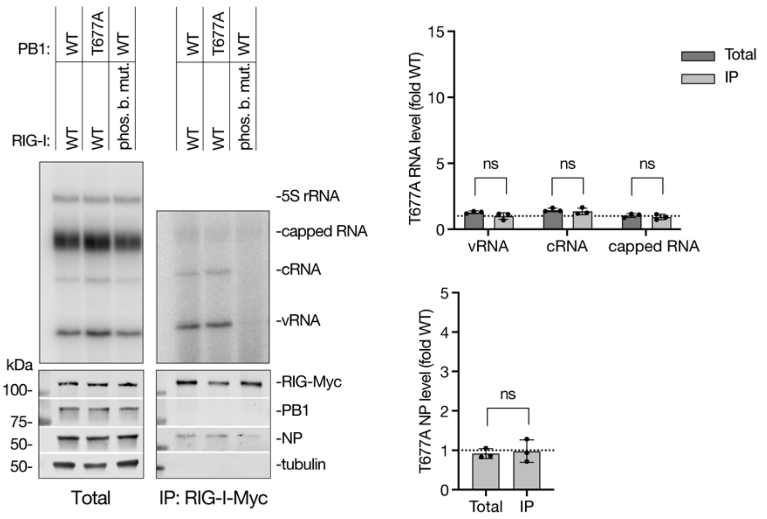

Supplementary Fig. 16| Immunoprecipitation of RIG-I in an RNP assay with segment 5. Myc-tagged RIG-I was co-expressed with wild-type or T677A mutant RNP and segment 5 (encoding NP), followed by immunoprecipitation. A phosphate-binding mutant of RIG-I was used as a negative control. RNA levels in total and immunoprecipitated (IP) fractions were measured by primer extension with internal +sense (NP 149+) and -sense (NP 149-) primers. Protein levels were assessed by western blotting. Shown are a representative image (left) and quantification from three independent experiments (mean ± SD) (right). Statistical significance was determined using two-way ANOVA with Šídák's multiple comparisons test (top right) or an unpaired two-tailed t-test (bottom right) (ns=non-significant).

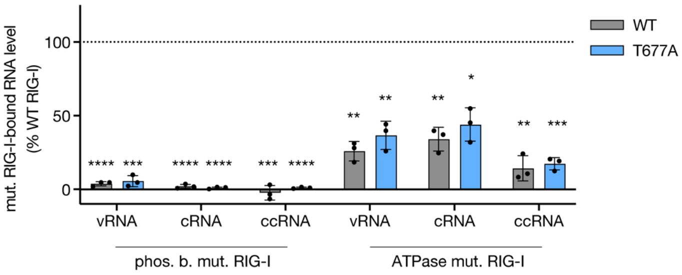

Supplementary Fig. 17| Quantification of RNA binding by RIG-I mutants. Immunoprecipitation of wild-type, phosphate-binding mutant, or ATPase mutant Myc-tagged RIG-I was performed in an RNP assay with a short NA196 template. RNA levels were quantified by primer extension assay with terminal cRNA and vRNA primers. Data are shown as (mean ± SD) from three independent experiments. Statistical significance was determined using one-sample t-test (*P<0.05, **P<0.01, ***P<0.001, ****P<0.0001).

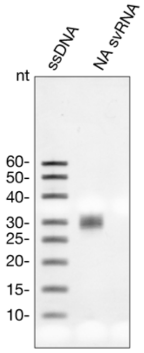

Supplementary Fig. 18| Generation of segment 6 (NA) svRNA by *in vitro* transcription. Bulk *in vitro* transcribed 25 nt-long svRNA was gel purified, and the RNA quality determined by denaturing PAGE, loading 100 ng per lane.

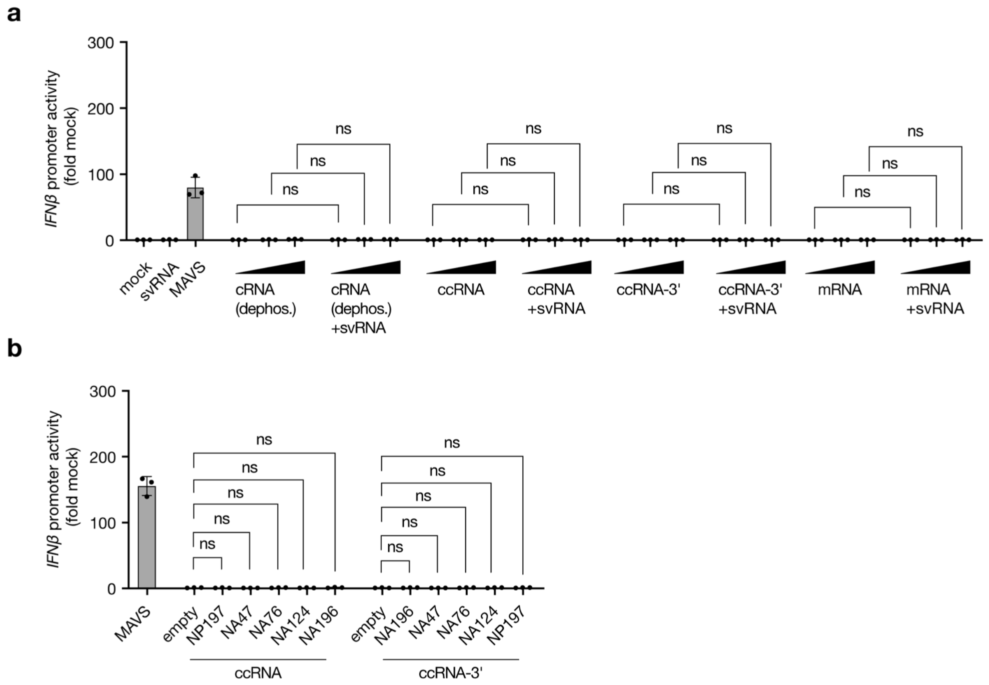

Supplementary Fig. 19| Immunostimulatory activity of ccRNA and ccRNA complexes with a complementary negative-sense RNA in *RIG-I* -/- HEK293-luc cells. a, Increasing concentrations (0.05, 0.1 and 0.2 pmol) of *in vitro* transcribed NA196-based cRNA (dephosphorylated), ccRNA, ccRNA-3', and mRNA molecules were co-transfected with 1 pmol of segment 6 svRNA into *RIG-I* -/- HEK293-luc cells. Total 293T RNA (untransfected) was used as a negative control and a MAVS-encoding plasmid was used as a positive control. *IFN-β* promoter activity was measured 20 hours post-transfection. **b**, As further controls, 0.2 pmol of *in vitro* transcribed NA196-based ccRNA or ccRNA-3' molecules, or an equivalent amount of total 293T (untransfected) RNA was co-transfected with pPolI plasmids encoding 47-196 nt long segment 6-based vRNA templates or a segment 5-based NP197 template into *RIG-I* -/- HEK293-luc cells. An empty plasmid was used as a negative control, while a MAVS-encoding plasmid was used as a positive control. *IFN-β* promoter activity was measured 20 hours post-transfection. Data are presented as mean ± SD from three independent experiments. Statistical significance was determined using two-way ANOVA with Šídák's multiple comparisons test; (ns=non-significant).

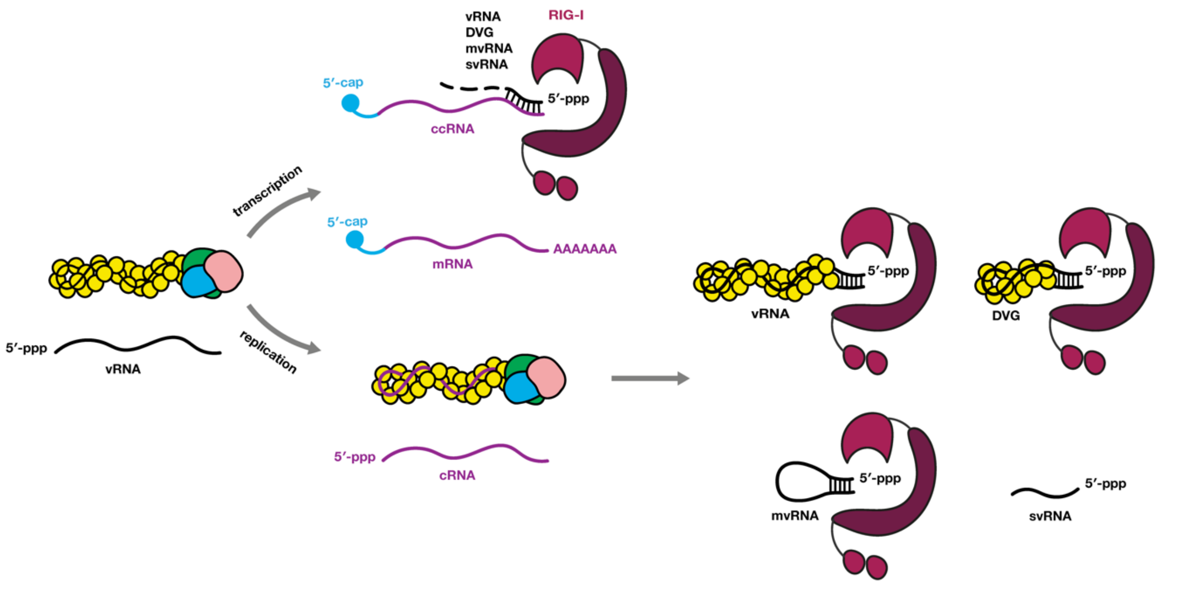

Supplementary Fig. 20| Model of RIG-I activation during IAV infection. RIG-I binds the partially complementary promoter of viral replication products, such as vRNA, DVG, and mvRNA molecules. In addition, RIG-I detects the perfectly complementary dsRNA formed by the hybridization of the 3' terminus of ccRNAs with the 5' triphosphorylated terminus of either vRNA, svRNA, DVG, or mvRNA molecules.
